# Streamlined Identification of Metallopeptides for Intracellular Catalysis Using Positionally Addressable Combinatorial Libraries

**DOI:** 10.1021/acscatal.5c00525

**Published:** 2025-05-08

**Authors:** Carmen González-González, Laura Martínez-Castro, Soraya Learte-Aymamí, Clara Pose-Insua, José R. Couceiro, Pau Martin-Malpartida, Maria J. Macias, Jean-Didier Maréchal, José L. Mascareñas, M. Eugenio Vázquez

**Affiliations:** a Centro Singular de Investigación en Química Biolóxica e Materiais Moleculares (CiQUS), Departamento de Química Orgánica, 16780 Universidade de Santiago de Compostela, Santiago de Compostela 15705, Spain; b Insilichem, Departament de Química, Universitat Autònoma de Barcelona, Cerdanyola 08193, Spain; c Centro Singular de Investigación en Química Biolóxica e Materiais Moleculares (CiQUS), 16780 Universidade de Santiago de Compostela, Santiago de Compostela 15705, Spain; d Institute for Research in Biomedicine (IRB Barcelona), The Barcelona Institute of Science and Technology, Baldiri Reixac, 10, Barcelona 08028, Spain; e Institució Catalana de Recerca i Estudis Avançats (ICREA), Passeig Lluís Companys 23, Barcelona 08010, Spain

**Keywords:** catalysis, peptides, combinatorial chemistry, computational chemistry, biocatalysis

## Abstract

The discovery and development of artificial catalysts to carry out bioorthogonal reactions in living cells is a primary goal at the interface of Chemistry and Biology. Current approaches rely on time-consuming trial-and-error methods. As an alternative, we show that positionally addressable combinatorial libraries (SPOT libraries) provide a significant advantage for the efficient identification of novel catalytic metallopeptides. Using these libraries, we were able to rapidly identify catalytic β-hairpin palladopeptides capable of promoting efficient depropargylation reactions in challenging intracellular environments.

## Graphical abstract

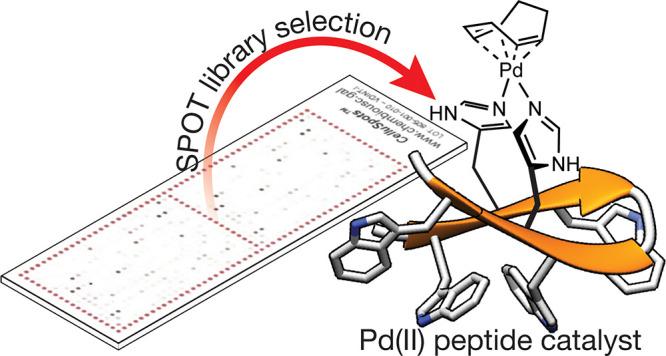

## 1 Introduction

The development of artificial organometallic catalysts that promote biocompatible reactions in living cells is a major research challenge in chemical biology that can unlock new opportunities to study and manipulate biological processes. However, despite some remarkable examples reported in the last couple of decades,^1–10^ organometallic catalysts typically show low efficiency inside cells due to the rapid deactivation of the metal active sites by reactive metabolites in the cytoplasm.^11–14^ A promising alternative to discrete metal catalysts is the use of artificial metalloenzymes containing late transition metals. However, assembling these complex structures inside cells remains a major challenge.^15–25^ Metallopeptides, halfway between conventional metal complexes and artificial protein-based catalysts, may hold the key to implementing organometallic transformations in biological settings.^26^ Peptides are large enough to fold into structures that protect their metal active site from poisoning by reactive metabolites and can even assist in the catalytic process^27^ yet small enough to efficiently cross cellular barriers and accumulate inside living cells.^28,29^ Furthermore, peptides are modular, so their structure and properties can be optimized using well-established engineering rules.^30–33^ Indeed, our group has shown that designed palladopeptides can efficiently internalize into mammalian cells and promote intracellular reactions.^34–36^ Despite these advantages, these catalytic metallopeptides still lag behind natural enzymes.^35^ Moreover, their rational design is time-consuming and does not guarantee optimal results.^37^ Combinatorial methods, which could be a more practical approach, have been frustrated by the obstacles associated with library screening to identify catalytic sequences.^38–46^

SPOT libraries, introduced in 1992,^47^ involve synthesizing peptides on planar matrices, typically cellulose sheets, by spotting the different reagents at defined positions on the solid support;^48–50^ the resulting array can be positionally addressed, thereby greatly simplifying the screening process.^48,51^ This simplicity has made SPOT peptide arrays popular in biology for the study of protein–protein interactions (i.e., epitope mapping, enzyme substrate analysis, antibody specificity, interactome profiling, etc.).^52–55^ Curiously, SPOT libraries have been largely ignored in catalysis, except for an isolated example where they were used to identify an organocatalytic peptide esterase.^56^

We envisioned that SPOT technology would be particularly useful for the discovery of catalytic metallopeptides because it provides access to reasonably sized libraries that can be efficiently screened using fluorogenic substrates. To implement this methodology, we focused on β-hairpin scaffolds because they are relatively simple while offering many opportunities to arrange pairs of chelating residues and fine-tune the second coordination sphere to modulate the catalytic properties of the system.^57–60^ In this study, we demonstrate the feasibility of this approach, which allows for the straightforward identification of a novel β-hairpin palladopeptide capable of catalyzing a depropargylation reaction in aqueous buffers and even in living mammalian cells.

## 2 Results and Discussion

As β-hairpin scaffold, we chose the tryptophan zipper (trpzip) family of peptides, which are short (12- or 16-residue), well-folded, monomeric β-hairpins, stabilized by interstrand stacking of Trp side chains.^61^ The NMR structures of several of these remarkable peptides have been determined, and the Trp zipper has been proven as a suitable scaffold for metal binding,^60^ which facilitates the structure-guided design of a focused combinatorial library of metallopeptides.^61^ Based on the original report of the trpzip family,^61^ we chose the trpzip1 dodecamer, S^1^WT^3^WE^5^ GN K^8^WT^10^WK^12^, as the basis of our library. This model peptide contains a central type β-II′ turn (E^5^ GN K^8^) flanked by two WTW strands that interlock with their Trp side chains to form the hydrophobic core of the hairpin. Our SPOT library was designed to preserve the central GN sequence that defines the β-turn as well as the Trp core as key structural elements, while scanning the positions 1, 3, 5, 8, 10, and 12 (x^1^Wx^3^Wx^5^ GN x^8^Wx^10^Wx^12^), all of which are oriented in the hairpin toward the concave side of the hairpin to facilitate a bidentate coordination to a Pd­(II) complex, while offering some steric protection to avoid a rapid deactivation in complex aqueous media (Figure 1a).^34^

**Figure 1:**
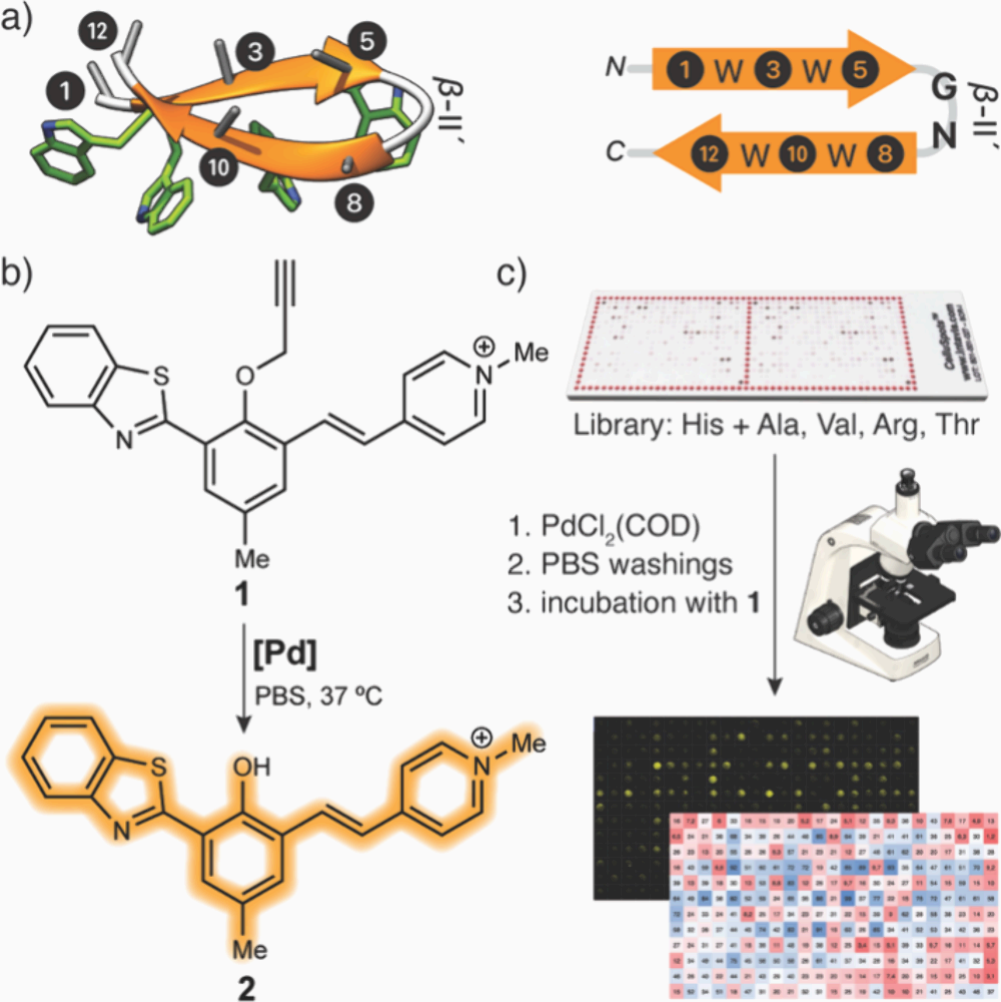
Scheme of screening of catalytic metallopeptides. (a) Left: Structure of a trpzip hairpin (PDB: 1LE0) indicating as stubs the positions mutated in the screening (1 to 12 from the *N*- to *C*-terminus), right: schematic representation of the trpzip hairpin indicating the positions of the screening. (b) Fluorescent readout and results of the screening of the probe **1**. (c) Screening of the library. The CelluSPOT slide was first incubated for 1 h with a 1 mM solution of PdCl_2_(COD), followed by a 10 min wash with PBS to remove any uncoordinated palladium. Next, the slide was treated with a 200 μM solution of the fluorogenic probe **1**, and the emission of the spots was recorded at 0, 4, and 24 h. The catalytic metallopeptides are identified based on their position in the array and their emission intensity, which correlates with the progress of the depropargylation reaction and formation of **2**.

The screening tested the 11 possible arrangements of two adjacent His in the β-hairpin trpzip (e.g., His^1^/His^3^, His^1^/His^12^, His^1^/His^10^, His^3^/His^12^, and so on), while randomizing in each case the four remaining residues. However, full site saturation mutagenesis with a subset of 14 proteinogenic residues—excluding His, Trp, and the potentially coordinating Cys, Met, Asp, and Glu residues—would still generate a prohibitively large library of more than 10^5^ peptides. Thus, we decided to run a simplified proof-of-concept screening using a minimal set of four amino acids (Ala, Val, Thr, Arg), which were selected on the basis of their β-sheet propensity,^62^ physicochemical properties, and performance in solid-phase peptide synthesis.^63^ This allowed us to build a simplified library of 264 peptides using a bespoke Python script that generates the combinations of the coordinating His residues and the permutations of selected amino acids for the remaining positions to generate the library sequences. Customization options for this tool include the selection of the number and type of coordinating residues, the positions for mutations, and the set of residues to be permutated (see the Supporting Information). Once synthesized, the resulting cellulose-supported SPOT library was transferred to a microscope slide using the CelluSPOT technique to miniaturize the screening assay.^54,64^

As catalytic reaction, we chose the depropargylation of fluorogenic probe **1**, which exhibits low emission when protected but becomes highly fluorescent upon depropargylation (Figure 1b). In brief, the CelluSPOT slides with the complete library were mixed with a solution of the palladium precursor dichloro­(1,5-cyclooctadiene) palladium­(II), PdCl_2_(COD), in aqueous buffer for 1 h to form the metallopeptides *in situ*, followed by extensive washing with PBS to remove any unbound, catalytically active, metal salt (see the Supporting Information for the selection of the palladium source, Figure S10).^34^ The array was then treated with the fluorogenic probe **1** (200 μM in PBS), and the depropargylation reaction progress was tracked by measuring the emission at 635 nm upon irradiation at 330 nm (Figure 1c and Supporting Information for a detailed screening protocol). The screening was run three times and demonstrated excellent reproducibility, particularly in identifying the most active and inactive sequences (standard deviation of 6.5% for the top 25 hits). Importantly, as a preliminary test of the catalytic metallopeptide robustness in complex aqueous media, the screening was also performed in Dulbecco’s modified Eagle medium (DMEM) with HEPES buffer, yielding qualitative results similar to those obtained in the PBS screenings.

An analysis of the frequency of the different residues at each position in the 10 best sequences^21^ revealed a distinct preference for the His residues at positions His^3^/His^10^ facing each other across the hairpin, followed by trivial peptides with two adjacent His residues in the C-terminal strand at positions His^10^/His^12^ (Figure 2a,b). Additionally, there was some tendency toward Val at position 5, while Thr was the least favored residue in most positions, except at the *N*- and *C*-termini where the hydrophobic and bulky Val was negatively selected (Figure 2b). Focusing on the hits with the His^3^/His^10^ combination, the standout feature was the selection of Arg at position 8, often correlated with Thr at position 1 and Val at position 5 (Figure 2c and residue correlation analysis in the Supporting Information, Figure S6).

**Figure 2:**
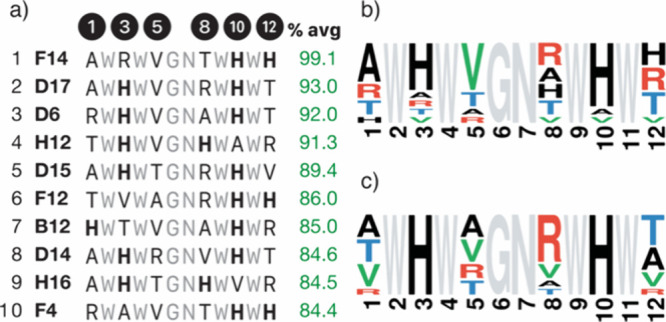
(a) Ten best catalytic sequences ranked by the relative amount of depropargylated product **2** after 24 h (green column, normalized average of the relative fluorescence emission intensity measured in three experiments). (b) Sequence logo of the 10 best hits of the CelluSPOT library.^65^ (c) Sequence logo of the 10 best peptides selected in the library screening that belong to the set with His residues in positions 3 and 10.

Following the screening experiments, two representative β-hairpin peptides were individually synthesized for physicochemical characterization and a detailed study of their coordinative and catalytic properties in solution. As one of the top hits of the His^3^/His^10^ family, we selected the peptide **D14**, *A* ^*1*^W**H** ^3^W*R* ^5^ GN VW**H** ^10^WT. As negative control, we selected peptide **D4**, *R* ^*1*^W**H** ^3^W*A* ^5^ GN VW**H** ^10^WT, which showed lower emission intensity in the CelluSPOT assay and has almost the same sequence as **D14**, with Ala^1^ and Arg^5^ in **D14** swapped their positions in **D4** (Arg^1^/Ala^5^).

To study the metal coordination of **D14** and **D4**, we mixed equimolar amounts of each peptide (500 μM) with PdCl_2_(COD) in H_2_O and then analyzed the mixture by MS at different mixing times. A major peak with M/z corresponding to the [Pd­(II)]**D14** metallopeptide was observed as early as 15 min after mixing, whereas the **D4/**PdCl_2_(COD) mixture shows only the MS peak corresponding to the free peptide **D4**, even after 24 h (Supporting Information, Figure S3). In agreement with this qualitative analysis, fluorescence titrations of **D14** and **D4** confirmed that **D14** exhibits significantly stronger affinity for Pd­(II) than **D4** (*K*_D_ = 1.0 μM, and *K*_D_ > 1 mM for **D14** and **D4**, respectively (Supporting Information, Figure S13). These results strongly suggest that the poor catalytic performance of **D4** in the SPOT screening reflects its inability to form stable palladium complexes. In agreement with this result, the circular dichroism spectrum of **D14**, which is dominated in the far-UV region by a positive exciton couplet arising from the electronic coupling between the indole side chains,^66^ changes significantly upon addition of PdCl_2_(COD), while the CD spectrum of **D4** remains largely unchanged in the presence of the same Pd source (Figure 3a).

**Figure 3:**
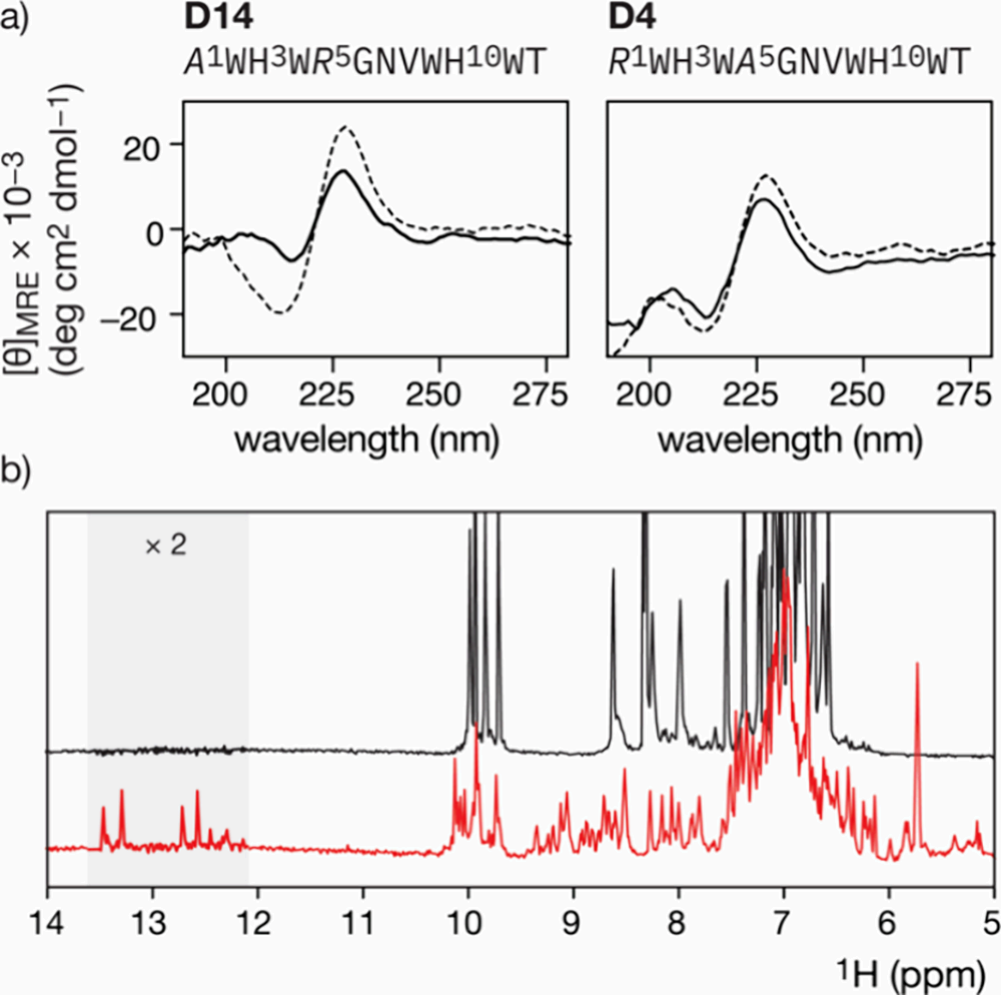
(a) Left: CD spectra of 5 μM **D14** (dashed line) and 5 μM **D14** in the presence of and 1 equiv of PdCl_2_(COD) (thick solid line). Right: CD spectra of 5 μM **D4** (dashed line) and 5 μM **D4** in the presence of and 1 equiv of PdCl_2_(COD) (thick solid line). Measurements were performed in 10 mM phosphate buffer at pH 7.5 and 100 mM NaCl using a 2 mm cell at 4 °C. (b) 1D NMR data of the free peptide **D14** (black) and in the presence of 4.0 equiv of Pd­(II). The His resonance region, which shows the metal coordination in the red trace, is highlighted in gray, and the intensity in that region magnified for clarity. Spectra were acquired using a Bruker Avance III 600 spectrometer at 295 K, operating at 600.23 MHz (^1^H frequency), equipped with a *z*-pulse field gradient unit and a triple (^1^H, ^13^C, ^15^N) resonance cryoprobe head.

To gain further insights into the metal coordination of **D14**, we acquired NMR data of the peptide by itself, and in the presence of PdCl_2_(COD). The 1D data corresponding to the free peptide at 1.0 mM concentration (Figure 3) shows a poor dispersion of the chemical shifts in the amide region (from 10 to 8 ppm), consistent with an extended conformation of the peptide. Remarkably, the addition of increasing metal concentrations induces the appearance of well-dispersed amide signals, and also of the characteristic chemical shifts of the histidine side chain resonating between 12 and 13.5 ppm upon Pd­(II) coordination. These signals indicate that the peptide is no longer flexible and extended but is adopting a defined secondary structure, consistent with the folding of the trpzip β-hairpin, as seen in other designed β-sheet metallopeptides.^67^ We did not observe peptide aggregation at the mM range of concentration as described for the TrpZip1 and related tryptophan zipper peptides (see Supporting Information Figure S12).^68–70^

To understand at the atomic level the different coordination properties of **D14** and **D4**, we first run DFT calculations considering *cis* or *trans* geometries through the δ or ε nitrogen atoms of the His side chains (see the Supporting Information for details). The relative stabilities of the different complexes revealed a slight preference for *cis* coordination and no significant differences in binding through δ or ε nitrogen atoms in the His imidazoles. However, the small energy difference between the structures—less than 5 kcal/mol—suggests that different coordination geometries could be favored by the conformational preferences of each peptide.

In agreement with the NMR experiments, molecular dynamics (MD) simulations of the free peptides showed that both **D14** and **D4** were highly dynamic (although **D14** was more likely to adopt a β-hairpin conformation than **D4**, as seen in the timelines in Figure 4a,c). In contrast, the trajectories and Ramachandran plots of the MD runs with the corresponding metallopeptides indicate that [Pd­(II)]**D14** adopts a stable β-hairpin conformation (once again, consistent with the NMR spectrum in Figure 3) while [Pd­(II)]**D4** appears strained and tends to adopt other conformations, suggesting that the geometrical constraints imposed by the Pd coordination are more compatible with the conformational preferences of **D14** than those of **D4** (Figure 4b,d); thus, [Pd­(II)]**D14** is expected to be a more stable metallopeptide. Gaussian accelerated molecular dynamics (GaMD) simulations, which perform a wider exploration of the conformational space by applying a potential boost to avoid confinement at local minima in the potential energy surface, provided qualitatively similar results.

**Figure 4:**
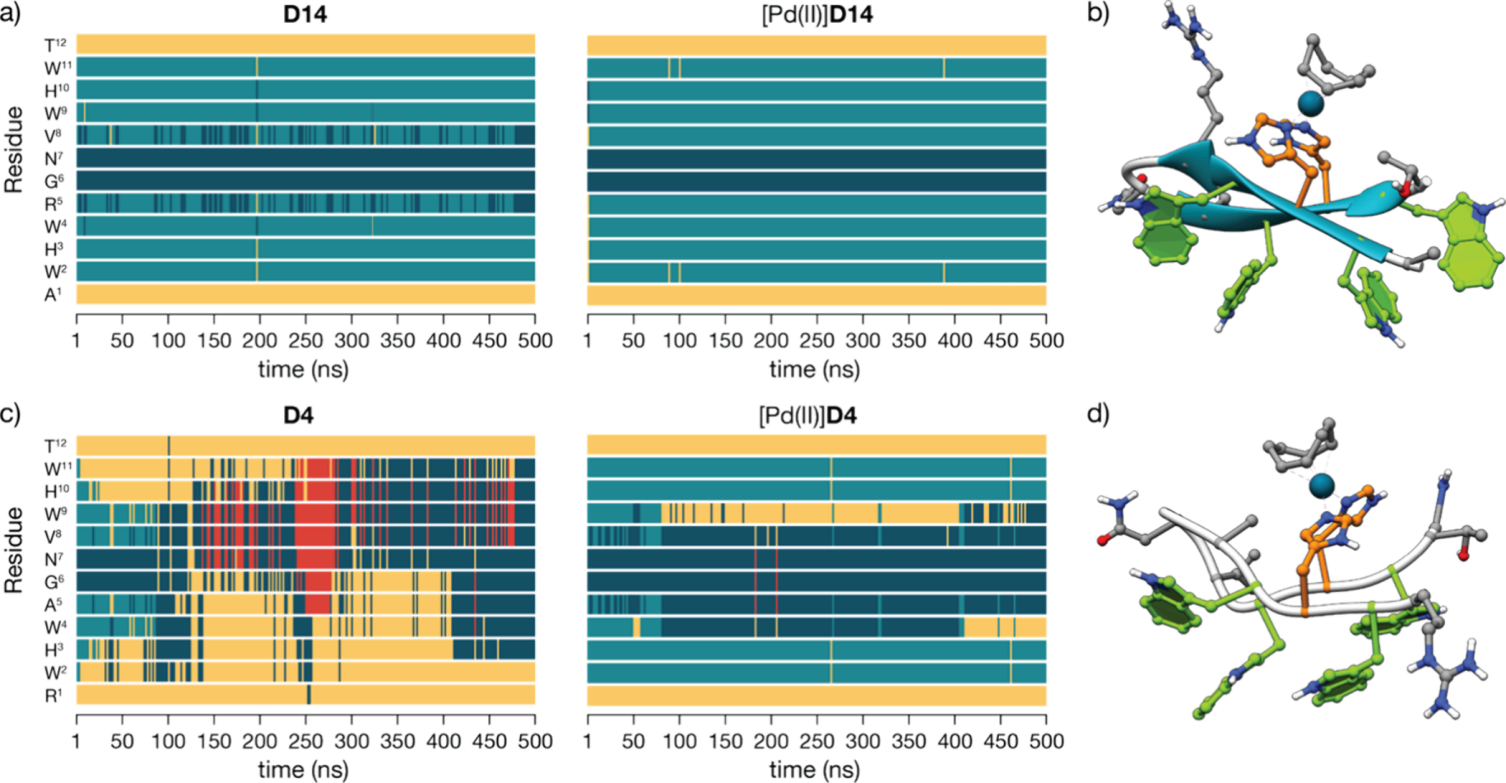
(a) Timeline of the MD simulations of **D14** (left) and [Pd­(II)]**D14** (right) showing the partial stabilization of [Pd­(II)]**D14**; yellow: disordered conformation; light blue: β-strand; dark blue: turn; red: helical conformation. (b) A representative structure of the major cluster in the MD simulation shows the folded hairpin with a well-folded hydrophobic core. (c) Timeline of the MD simulations of **D4** (left) and [Pd­(II)]**D4** (right) showing the highly flexible **D4** β-hairpin that is only marginally stabilized by the formation of the complex [Pd­(II)]**D4**. Same color coding as before for the different conformations. (d) Structure of the most populated cluster of the MD displaying the poorly folded β-hairpin structure.

Having confirmed the formation of the palladopeptide [Pd­(II)]**D14** in solution, we tested the catalytic depropargylation reaction *in vitro* by mixing a 200 μM solution of **1** in PBS with 10 mol % of the preformed catalyst [Pd­(II)]**D14**, obtained by mixing stoichiometric amounts of **D14** and PdC_l2_(COD) in water. HPLC-MS analysis of the crude reaction product using an internal standard showed that the desired depropargylation product was formed in 56% yield after 24 h. The reaction can also be catalyzed in biologically relevant media such as in Dulbecco’s modified eagle medium (DMEM), although this requires a higher concentration of the [Pd­(II)]**D14** catalyst (50 mol %), resulting in a moderate yield of 26%. In contrast, when using peptide **D4**, which has a lower binding affinity for Pd­(II), the yield of the depropargylation was significantly reduced to 16%, similar to the yield obtained when using the discrete palladium complex PdCl_2_(COD) in the absence of peptide ligands (Figure S11).

To further validate the robustness of the CelluSPOT assay for identifying catalytic metallopeptides, we tested additional sequences that ranked high in the screening (peptides **F14** and **E10**, with high relative fluorescence intensities of 99.1 and 82.8, respectively), and isomeric control sequences that displayed poor induction of the depropargylation reaction (**F13**, and **E9**, with average emission of 21.2 and 8.8). As expected, **F14** and **E10** efficiently promoted the depropargylation reaction of **1** in DMEM (yields of 28 and 33% after 24 h), while the control sequences that performed worse in the screening, **F14** and **E10**, produced significant lower yields under the same conditions (18 and 16%, respectively), in line with the yield obtained when using PdCl_2_(COD) alone, confirming that this is the catalytic species responsible for the depropargylation (Figure S11).

With these results in hand, we were ready to investigate the viability of these palladopeptide catalysts in cells. To this end, we first studied the internalization of peptides **D14** and **D4** by fluorescence microscopy, which required the synthesis of N-terminal TAMRA-tagged peptides, **T-D14** and **T-D4** (Supporting Information). Incubation of the apo peptides with HeLa cells revealed rather poor internalization properties in both cases. Remarkably, preincubation of **T-D14** with 1 equiv of PdCl_2_(COD) resulted in the appearance of bright intracellular emission (Figure 5a and Figure 5b, respectively). No intracellular fluorescence was observed with **T-D4** either by itself, or in the presence of PdCl_2_(COD) (Figures 5c and 4d). Therefore, it seems that the palladium stapling enhances the cellular uptake, likely through stabilizing a compact structure.^34,71–73^ ICP-MS analysis of cellular extracts revealed that the palladium content in cells treated with the preformed palladopeptide [Pd­(II)]**T-D14** is significantly higher than that of the mixture of **T-D4** and PdCl_2_(COD) or with PdCl_2_(COD) itself (Figure 5e).

**Figure 5:**
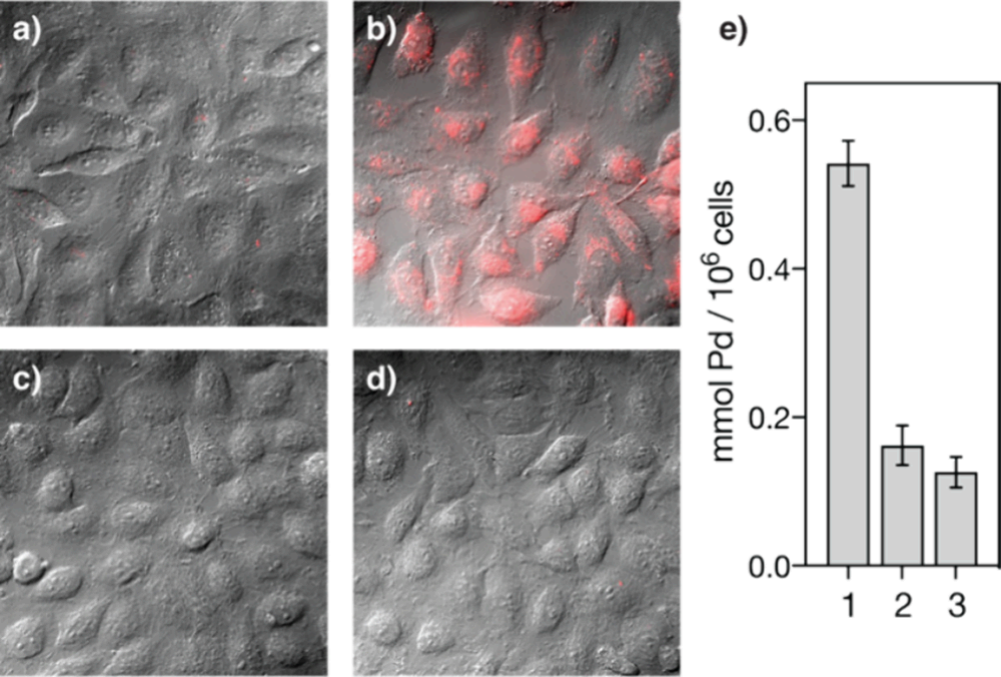
Cellular uptake of **T-D14** and **T-D4** in HeLa cells. Left: fluorescence micrographs recording the emission at 579 nm upon irradiation at 546 nm of (a) 50 μM **T-D14**, (b) 50 μM **T-D14** and PdCl_2_(COD), (c) 50 μM **T-D4**, (d) 50 μM T-**D4** and PdCl_2_(COD), (e) quantification of intracellular palladium by ICP-MS. Bars 1–3: 1, **T-D14** with PdCl_2_(COD); 2, **T-D4** with PdCl_2_(COD); 3, PdCl_2_(COD).

The good internalization of [Pd­(II)]**T-D14** encouraged us to study its catalytic properties in living cells. Mammalian HeLa cells were incubated with a 50 μM solution of probe **1** in FBS-DMEM for 1 h at 37 °C. The medium was removed, and the cells were washed twice with FBS-DMEM to remove any extracellular probe residues. The cells were then treated with a 50 μM solution of the preformed catalyst [Pd­(II)]**D14** in fresh media for 1.5 h, followed by two washes with PBS before being imaged by wide-field fluorescence microscopy. Gratifyingly, we observed a very intense intracellular emission arising from product **2** indicating an efficient depropargylation reaction (Figure 6a). As expected, no emission was observed when the same experiment was performed with a mixture of **D4** and PdCl_2_(COD) (Figure 6b). Furthermore, in contrast to the in vitro results, control experiments showed that PdCl_2_(COD) does not generate any intracellular fluorescence by itself, supporting the role of the peptide in protecting the catalytic center (Supporting Information, Figure S7). The formation of the expected product **2** was further confirmed by HPLC-MS analysis of methanolic extracts of the cells after the reaction. By using appropriate calibration curves, we were able to quantify the depropargylation product generated inside the cells, confirming that the reaction is efficient only in the presence of palladopeptide [Pd­(II)]**D14** (Figure 6c).

**Figure 6:**
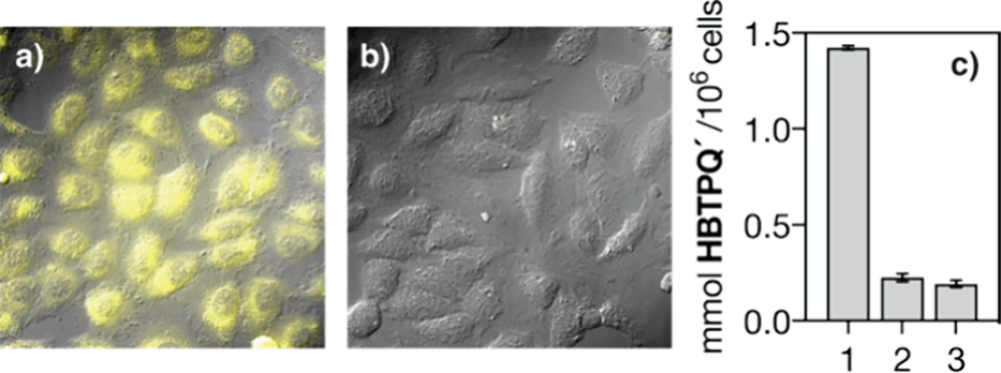
Intracellular depropargylation of **1** was monitored by fluorescence microscopy in HeLa cells. (a) Incubation with the preformed palladopeptide obtained by mixing **D14** and PdCl_2_(COD). (b) Incubation with the mixture of **D4** and PdCl_2_(COD). Incubations were made in FBS-DMEM. Bright-field images are superimposed to the orange emission channel recorded at 515–700 nm upon excitation at 385 nm. (c) Intracellular formation of **1** measured by HPLC-MS analysis of metabolic extracts. Bars 1–3:1, **D14** with PdCl_2_(COD); 2, **D4** with PdCl_2_(COD); 3, PdCl_2_(COD).

By combining the concentration of intracellular palladium obtained by ICP-MS with the concentration of the product, we were able to calculate a turnover number about 5 for the depropargylation reaction catalyzed by [Pd­(II)]**D14**, which is modest but confirms the existence of catalytic cycles. The toxicity of peptide **D14**, palladopeptide [Pd­(II)]**D14**, and PdCl_2_(COD) salt was tested by MTT assays in HeLa cells after 24 h of incubation, showing about 90% viability at 50 μM (the highest concentration used in our assays). Interestingly, [Pd­(II)]**D14** systematically shows less toxicity than PdCl_2_(COD) at all concentrations, suggesting that the [Pd­(II)]**D14** metallopeptide is rather stable in the cytoplasm, which is in line with the observed (relative) stability of the catalytic species in solution that allows several catalytic cycles before deactivation.

## 3 Methods

### SPOT Libraries

Spot peptide libraries and CelluSPOT slides were synthesized by *Intavis Peptide Services GmbH*, Waldhäuser Straße 64, 72076 Tübingen, Germany, following reported procedures in the literature. The following protocol was followed to carry out screening of the SPOT library: The CelluSPOT slide was incubated for 1 h with agitation with 10 mL of a 1 mM solution of dichloro­(1,5-cyclooctadiene) palladium­(II)—PdCl_2_(COD)—the slide is washed three times with PBS for 10 min per wash to remove any uncoordinated palladium. Next, 50 μL of a 200 μM solution of the probe **1** is added, the slide is covered with a coverslip, and the edges are sealed with *cytosyl*. In the negative control, the procedure is the same except that plain water is added in the coordination step. The slide is taken to the microscope, and the emission of the spots is recorded at 0, 4, and 24 h. The plate was kept in a humid atmosphere while the studies were being carried out under a microscope to prevent it from drying out.

### Solid-Phase Peptide Synthesis and On-Resin TMR Tagging

All peptide synthesis reagents and amino acid derivatives were from *Sigma-Aldrich* and *Iris Biotech*; amino acids were purchased as protected Fmoc amino acids with the standard side-chain protecting scheme: Fmoc-Ala-OH, Fmoc-Val-OH, Fmoc-Arg­(Pbf)-OH, Fmoc-Trp­(Boc)-OH, Fmoc-Thr­(*t-*Bu)-OH, and Fmoc-His­(Trt)-OH. Peptides were synthesized on an H-Rink-Amide ChemMatrix (0.57 mmol/g loading) from *Biotage AB.* Peptides were synthesized following standard Fmoc-peptide synthesis protocols on a 0.1 mmol scale using a 0.5 mmol/g loading *H-Rink* amide *ChemMatrix* resin with a *Liberty Lite* automatic microwave assisted using a peptide synthesizer from CEM Corporation. The amino acids were coupled in 5-fold excess DIC as the activator, Oxime as base, and DMF as solvent. Couplings were conducted for 4 min at 90 °C. Deprotection of the Fmoc-protecting group was performed by treating the resin with 20% piperidine in DMF for 1 min at 75 °C. **TMR coupling**: For **T-D14** and **T-D4** after the N-terminal amino acid, we coupled Fmoc-6-aminohexanoic acid (Fmoc-Ahx-OH) as the spacer between the peptide, and after removal of the Fmoc-protecting group, a mixture of 5-carboxytetramethylrhodamine (0.15 mmol, 64.5 mg, 3 equiv), 3 equiv of HATU, and 5 equiv of DIEA 0.2 M in DMF was added onto the peptide resin and mixed with nitrogen bubbling for 60 min. A **cleavage/deprotection step** was performed by treatment of the resin-bound peptide for 2 h with the following cleavage cocktail: 900 μL of TFA, 50 μL of CH_2_Cl_2_, 25 μL of H_2_O, and 25 μL of TIS (1 mL of cocktail for every 40 mg of resin). The resin was filtered, and the cleavage mixture was added onto ice-cold diethyl ether. After 10–30 min, the precipitate was centrifuged and washed again with of ice-cold ether. The solid residue was dried under argon and redissolved in water. **Purification of the peptides** was performed on a semipreparative RP-HPLC with an *Agilent* 1100 series LC equipped with a UV–visible detector using a *Phenomenex Luna-C18* (250 × 10 mm) reverse-phase column. Standard conditions for purification by RP-HPLC consisted of a linear gradient 5 to 75% B over 40 min at a flow rate of 4 mL/min (A: H_2_O 0.1% TFA, B: CH_3_CN 0.1% TFA). Collected fractions with pure products were lyophilized with a *ThermoSavant Modulyo D* lyophilizer equipped with an *Edwars RV* high-vacuum pump.

### Computational Studies

The optimization of the Pd-coordinated intermediates was carried out at the DFT level of theory using Gaussian 16. For the Pd, the B3LYP functional was used combined with the SDD pseudopotential and its associated double-ζ bases set as well as a set of *f* polarization functions; for the rest of the atoms, the same functional with the 6-31G­(d) basis set was used, adding GD3 correction for dispersion. Solvent was included with the solvent-polarizable dielectric continuum model (SMD) in water. Harmonic frequency calculations were performed with tight convergence to ensure that the right vibrational modes were found. The **classical molecular dynamics** simulations were carried out with Amber20, using the Amber14SB force field for the protein, and the force constants and equilibrium parameters derived for Pd (from the aforementioned QM calculations) with the Seminario method. Point charges of Pd coordinated to both His were derived using the RESP (restrained electrostatic potential) model. Finally, force field building was performed with MCPB.py. The trajectories were set up with *xleap* (from AmberTools20), introducing each system in a cubic box of solvate TIP3P water molecules and Cl^–^ ions (*ions1lm_126_tip3p.lib*) to equilibrate charges. First, the (metallo)­peptide is submitted to 3000 steps of minimization, and then 100 ps of equilibration at constant volume followed by 500 ps of equilibration at constant pressure, and finally 500 or 800 ns of production, all run with Amber20. Analysis of the trajectories was carried out with the MDTraj library for Python. The specific tools used include RMSD analysis along the trajectory, all-to-all RMSD analysis comparing each frame to the initial position, and PCA and cluster counting to assess convergence of the simulation. Ramachandran plots were created using data from *RamachanDraw* for the density estimates; φ and ψ angles from the whole trajectory were extracted with chimera. All plots were created with Matplotlib library for Python.

### Cell Internalization Studies

Cells were seeded on glass-bottom plates 48 h before treatment. Culture medium was removed, and DMEM containing 5% fetal bovine serum (FBS-DMEM) and peptides (5 μM) or metallopeptides (5 μM) were added. Before the addition to cells, peptides were preincubated with metal complexes (1:1) in water for 10 min. After 30 min, cells were washed twice with PBS and replaced with fresh FBS-DMEM to observe under the microscope with adequate filters. Digital pictures of the different samples were taken under identical conditions of gain and exposure.

### Intracellular Reactions

HeLa cells were seeded on glass-bottom plates 48 h before treatment. Culture medium was removed, and FBS-DMEM containing probe **1** (50 μM) was added. After 1 h of incubation, cells were washed twice with FBS-DMEM and a solution of metal complexes or metallopeptides (peptides were preincubated with metal complexes (1:1) in water for 10 min before the addition to cells) in FBS-DMEM were added. After a 1 h incubation, cells were washed twice in PBS and replaced with fresh FBS-DMEM to observe under the microscope with adequate filters. Digital pictures of the different samples were taken under identical conditions of gain and exposure.

## Conclusions

In conclusion, we have demonstrated the potential of SPOT libraries for the identification of catalytic metallopeptides. In particular, we have found a β-hairpin palladopeptide, [Pd­(II)]**D14**, that catalyzes a depropargylation reaction in vitro and even inside living mammalian cells. While the efficiency of the catalysis is far from that of natural metalloenzymes, the resulting systems can be considered as a miniature proto-metalloenzymes capable of working inside cells because of their good stability and cell internalization properties. These results lay the groundwork for further application of spatially addressable arrays with to discover efficient biorthogonal catalysts for the selective release of prodrugs using Trp zipper β-hairpins,^7,8^ or even for the screening of alternative small peptide scaffolds, such as the Trp cage,^74^ or *de novo* peptides.^75,76^

## Supplementary Material



## Data Availability

The library design script (lib.py), and the starting points and parameters for the MD studies are deposited in the CORA repository: 10.34810/data2018.

## Notes

Detailed peptide synthesis and characterization; synthesis of the fluorogenic probe 1; library sequences; screening of the SPOT library; residue correlation studies; computational studies; and PCA of the results (PDF)

## Abbreviations

trpzip: tryptophan zipper
COD: 1,5-cyclooctadiene
PBS: phosphate-buffered saline
FBS: fetal bovine serum
DMEM: Dulbecco’s modified Eagle medium
TAMRA: 5-carboxytetramethylrhodamine
