# Supplementary material for: Streamlined Identification of Metallopeptides for Intracellular Catalysis Using Positionally Addressable Combinatorial Libraries
Source: ACS Catal. 2025 May 8;15(10):8624–32. doi: 10.1021/acscatal.5c00525 (PMC12128173; doi:10.1021/acscatal.5c00525)
Supplement: Supplementary file 1 [file cs5c00525_si_001.pdf]

Supporting Information

Streamlined Identification of Metallopeptides for Intracellular Catalysis Using Positionally-Addressable Combinatorial Libraries

Carmen González-González,[a,§] Laura Martínez-Castro,[b,§] Soraya Learte-Aymamí,[a,§] Clara Pose-Insua,[a] José R. Couceiro,[c] Pau Martin-Malpartida,[d] Maria J. Macias,[d,e] Jean-Didier Maréchal,*[b] José L. Mascareñas,*[a] M. Eugenio Vázquez*[a]

[a] Centro Singular de Investigación en Química Biolóxica e Materiais Moleculares (CiQUS), Departamento de Química Orgánica, Universidade de Santiago de Compostela. Santiago de Compostela 15705, Spain.

[b] Insilichem, Departament de Química, Universitat Autònoma de Barcelona, Cerdanyola 08193, Spain.

[c] Centro Singular de Investigación en Química Biolóxica e Materiais Moleculares (CiQUS), Universidade de Santiago de Compostela. Santiago de Compostela 15705, Spain.

[d] Institute for Research in Biomedicine (IRB Barcelona), The Barcelona Institute of Science and Technology, Baldiri Reixac, 10, Barcelona 08028, Spain.

[e] Institució Catalana de Recerca i Estudis Avançats (ICREA), Passeig Lluís Companys 23, Barcelona 08010, Spain.

Abbreviations

DIC: *N,N′*-Diisopropylcarbodiimide; DIEA: *N*,*N*-diisopropylethylamine; HATU: 2-(1H-7-aza- benzotriazol-1-*yl*)-1,1,3,3-tetramethyluronium hexafluorophosphate; TFA: trifluoroacetic acid; TIS: triisopropylsilane; TMR: tetramethyl rhodamine

General Information

Procedures for the synthesis of precursors were performed under an atmosphere of dry nitrogen using vacuum-line and standard Schlenk techniques. Dry solvents were directly purchased from *Sigma Aldrich* and used without further purification. Chemical were purchased from *Sigma Aldrich*, *Alfa Aesar*, and *Strem* and used without further purification.

Concentration refers to the removal of volatile solvents via distillation using a rotary evaporator *Büchi R-210* equipped with a thermostatic bath *B-491*, a vacuum regulator *V-850*, followed by residual solvent removal under high vacuum.

All catalytic reactions were carried out without extra precautions to eliminate moisture or oxygen and open to air. Reaction mixtures were stirred using Teflon-coated magnetic stir bars. The abbreviation “rt” refers to reactions carried out approximately at 23 °C. Temperature was maintained using Thermowatch-controlled heating blocks. Thin-layer chromatography (TLC) was performed on silica gel plates (*Merck* 60 silica gel F_{254}) and components were visualized by observation under UV light and / or by treating the plates with $KMnO_4$ or *p*-anisaldehyde followed by heating. Flash chromatography was carried out on silica gel (*Merck* Geduran Su 60, 40 - 63 μM silica gel, normal phase). Dryings were performed with anhydrous $MgSO_4$.

^{1}H NMR (300 MHz and ^{13}C NMR (75 MHz) spectra were recorded at room temperature on a *Varian Mercury* 300 MHz spectrometer or at 295K in a Bruker Avance III 600 spectrometer, operating at 600.23 MHz (1H frequency), equipped with a z-pulse field gradient unit and a triple (^{1}H, ^{13}C, ^{15}N) resonance cryoprobe head. ^{13}C NMR (126 MHz) were recorded on a *Bruker DRX-500* spectrometer. The chemical shifts for protons (δ) are reported in parts per million downfield from tetramethylsilane and are referenced to residual protium in the NMR solvent ($CHCl_3$ δ = 7.26). Chemical shifts for carbon are reported in parts per million downfield from tetramethylsilane and are referenced to the carbon resonances of the solvent ($CDCl_3$ δ = 77.0). NMR spectra were analyzed using *MestreNova NMR* data processing software (www.mestrelab.com).

LC-MS analysis were carried out using *Bruker AmaZon IT-MS* with C18 column.

Fluorescence measurements were performed using a *Varian Cary Eclipse* fluorimeter thermostated cell compartment at 20 °C using 1 cm quartz cells. The measurements were made with the following settings: increment 1.0 nm, averaging time 0.1 s, excitation slit width 5.0 nm, emission slit width 10.0 nm, PMT voltage 700 V. UV-VIS measurements were made in a *Jasco V-630* spectrophotometer coupled to a *Jasco ETC-717* temperature controller, using a standard *Hellma* semi-micro cuvette (108.002-QS) with a light path of 10 mm.

Measurements were made at 20 °C. Acquisition parameters were: 220-700 nm range, scan speed of 200 nm/min, resolution of 0.2 nm.

SPOT libraries

Spot peptide libraries and CelluSPOT slides were synthesized by *Intavis Peptide Services GmbH*, Waldhäuser Straße 64, 72076 Tübingen, Germany following reported procedures in the literature. The following protocol was followed to carry out screening of the SPOT library: The CelluSPOT slide was incubated for 1 h with agitation with 10 mL of a 1 mM solution of dichloro(1,5-cyclooctadiene) palladium(II)—($PdCl_2$(COD). The slide is the washed 3 times with PBS for 10 min per wash to remove any uncoordinated palladium. Next, 50 µL of a 200 µM solution of the probe **1** is added, the slide is covered with a coverslip and the edges are sealed with cytosyl. In the negative control the procedure is the same except that only water is added in the coordination step. The slide is taken to the microscope and the emission of the spots is recorded at 0, 4, and 24 h. The plate was kept in a humid atmosphere while the studies were being carried out under the microscope to prevent it from drying out.

Solid-phase peptide synthesis and on-resin TMR tagging

All peptide synthesis reagents and amino acid derivatives were from *Sigma Aldrich* and *Iris Biotech*; amino acids were purchased as protected Fmoc amino acids with the standard side chain protecting scheme: Fmoc-Ala-OH, Fmoc-Val-OH, Fmoc-Arg(Pbf)-OH, Fmoc-Trp(Boc)-OH, Fmoc-Thr(*t*-Bu)-OH, Fmoc-His(Trt)-OH. Peptides were synthesized on a H-Rink-Amide ChemMatrix (0.57 mmol/g loading) from *Biotage AB.*

Peptides were synthesized following standard Fmoc-peptide synthesis protocols on a 0.1 mmol scale using a 0.5 mmol/g loading *H-Rink* amide *ChemMatrix* resin with a *Liberty Lite* automatic microwave assisted using peptide synthesizer from CEM Corporation. The amino acids were coupled in 5-fold excess DIC as activator, Oxime as base and DMF as solvent. Couplings were conducted for 4 min at 90 °C. Deprotection of the Fmoc protecting group was performed by treating the resin with 20% piperidine in DMF for 1 min at 75 °C.

TMR coupling: For T-**D14** and T-**D4** after the N-terminal amino acid, we coupled Fmoc-6-aminohexanoic acid (Fmoc-Ahx-OH) as spacer between the peptide and after standard removal of the Fmoc protecting group, a mixture of 5-carboxytetramethylrhodamine (0.15 mmol, 64.5 mg, 3 eq.), 3 eq. of HATU, and 5 eq. of DIEA 0.2 M in DMF was added onto the peptide resin and mixed with nitrogen bubbling for 60 min.

Cleavage/deprotection step was performed by treatment of the resin-bound peptide for 2h with the following cleavage cocktail: 900 µL TFA, 50 µL CH_2Cl_2, 25 µL H_2O and 25 µL TIS (1 mL of cocktail for every 40 mg of resin). The resin was filtered, and the cocktail was added onto ice-cold diethyl ether. After 10 - 30 min, the precipitate was centrifuged and washed again with of ice-cold ether. The solid residue was dried under argon and re-dissolved in water.

Purification of the peptides was performed on a semipreparative RP-HPLC with an *Agilent* 1100 series LC equipped with a UV-visible detector using a *Phenomenex Luna–C18* (250 × 10 mm) reverse-phase column. Standard conditions for purification by RP–HPLC consisted in a linear gradient 5 to 75% B over 40 min at a flow rate of 4 mL/min. (A: H_2O 0.1% TFA, B: CH_3CN 0.1% TFA). Collected fractions with pure products were lyophilized with a *ThermoSavant Modulyo D* lyophilizer equipped with an *Edwars RV* high vacuum pump.

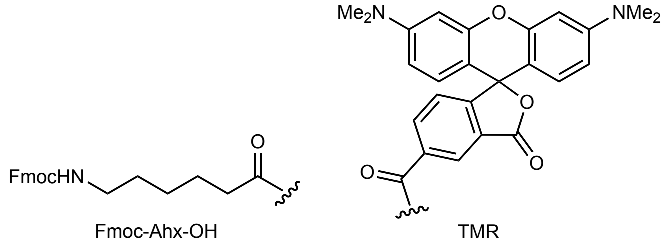

Figure S1. Structures of Fmoc-Ahx-OH, and TMR.

UHPLC-MS of the peptides

The compounds were analyzed by analytical UHPLC-MS with an *Agilent 1200* series LC/MS using a *SB C18* (1.8 µm, 2.1 × 50 mm) analytical column from *Phenomenex*. Standard conditions for analytical UHPLC consisted on a linear gradient from 5 to 95% of solvent B for 20 min at a flow rate of 0.35 mL/min (A: water with 0.1% TFA, B: acetonitrile with 0.1% TFA). Compounds were detected by UV absorption at 222, 270, and 330 nm. Electrospray Ionization Mass Spectrometry (ESI/MS) was performed with an *Agilent 6120*

Quadrupole LC/MS model in positive scan mode using direct injection of the purified peptide solution into the MS detector.

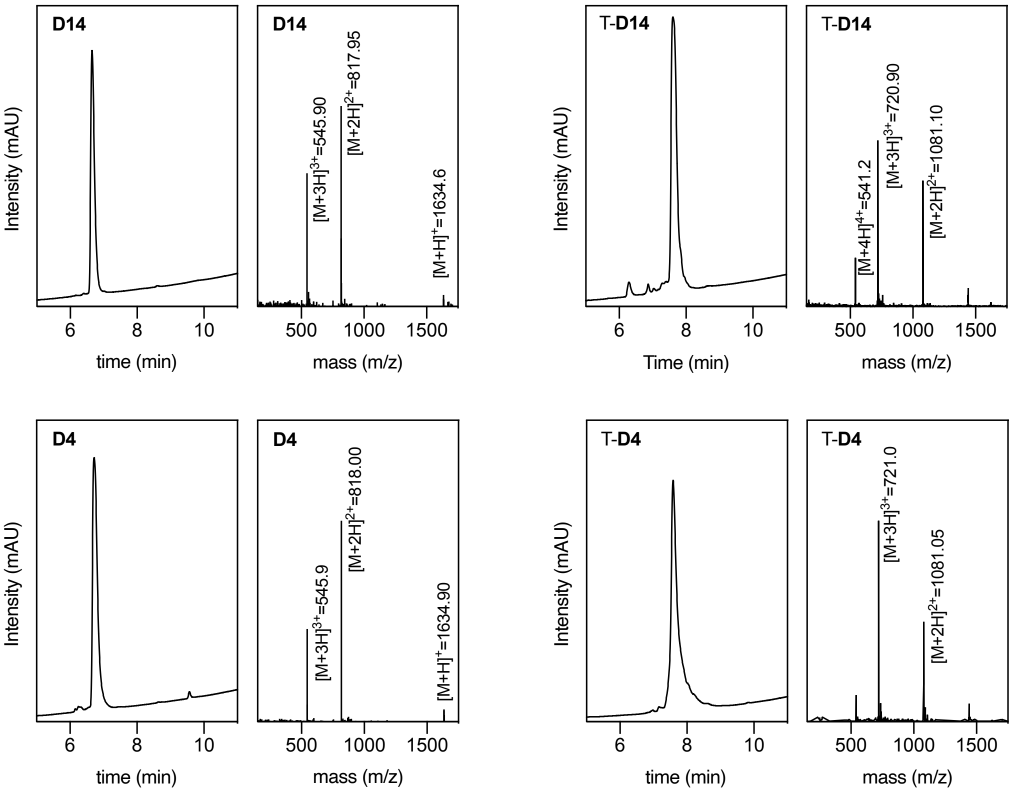

Figure S2. HPLC-EM-ESI+ (m/z) of purified peptides. 5→95% B over 30 min. **D14**, calcd. for $C_{80}H_{99}N_{25}O_{14}$: 1634.83, found: $[M+3H]^{3+}$ = 545.90, $[M+2H]^{2+}$ = 817.95; $[M+H]^{+}$ = 1634.9; T-**D14**, calcd. for $C_{111}H_{132}N_{28}O_{19}$: 2161.02, found: $[M+4H]^{4+}$ = 541.2 $[M+3H]^{3+}$ = 720.90, $[M+2H]^{2+}$ = 1081.10; **D4**, calcd. for $C_{80}H_{99}N_{25}O_{14}$: 1634.83, found: $[M+3H]^{3+}$ = 545.90, $[M+2H]^{2+}$ = 818.00; $[M+H]^{+}$ = 1634.9; T-**D4**, calcd. for $C_{111}H_{132}N_{28}O_{19}$: 2161.02, found: $[M+4H]^{4+}$ = 541.2 $[M+3H]^{3+}$ = 721.00, $[M+2H]^{2+}$ = 1081.05.

UHPLC-MS of the metallopeptides

Peptides **D4** and **D14** (1 mM) were mixed with equimolar amounts of $PdCl_2$(COD) in H_2O and the resulting mixtures were analyzed after 15 min. incubation by Electrospray Ionization Mass Spectrometry (ESI/MS) with an *Agilent 6120 Quadrupole* LC/MS model in positive scan mode using direct injection of the purified peptide solution into the MS detector. The peaks corresponding to the apo-peptides are shown in black, and those corresponding to the **D14** metallopeptide in pink.

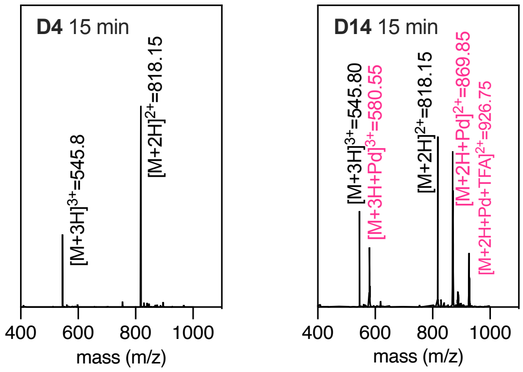

Figure S3. EM-ESI+ (m/z) **D4**, calcd. for $C_{80}H_{99}N_{25}O_{14}$: 1634.83; found: $[M+3H]^{3+}$ = 545.8, $[M+2H]^{2+}$ = 818.15, $[M]^{+}$ = 1634.75; **D14**, calcd. $C_{80}H_{99}N_{25}O_{14}Pd$: 1740.83; found: $[M+3H]^{3+}$ = 545.85, $[M+3H+Pd]^{3+}$ = 580.85, $[M+2H]^{2+}$ = 818.05, $[M+2H+Pd]^{2+}$ = 869.80, $[M+Pd+TFA]^{2+}$ = 927.1

Synthesis and characterization of 1

Synthesis. Synthesis of **1** was made following reported procedures.[1]

Scheme S1. Synthesis of propargylated probe (**1**).

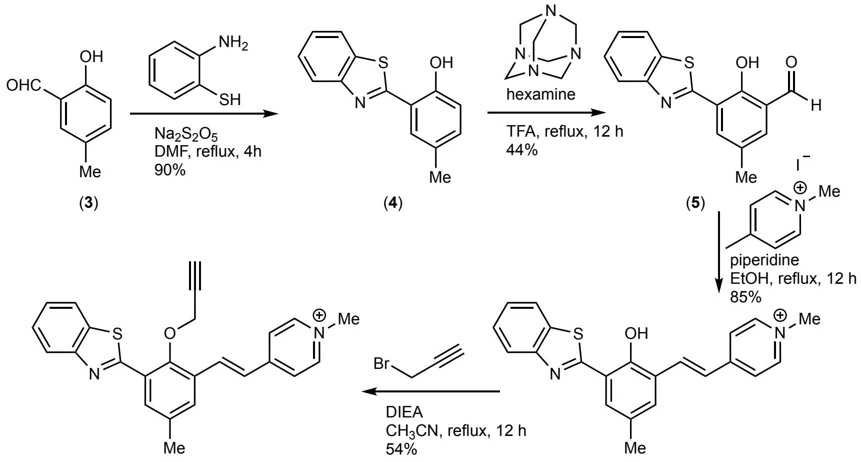

2-(benzo[d]thiazol-2-*yl*)-4-methylphenol. 2-hydroxy-5-methylbenzaldehyde (1.50 g, 11.0 mmol, 1.1 equiv.) and $Na_2S_2O_5$ (1.80 g, 9.5 mmol, 1.0 equiv.) were dissolved in 15 mL anhydrous DMF in a Schlenk flask under an inert atmosphere. 2-aminobenzenethiol (1.20 ml, 11.0 mmol, 1.1 equiv.) was added, and the reaction was then stirred at 110 °C for 3 h. 10 mL mili-Q water were added to the reaction mixture leading to the formation of a white precipitate. The precipitate was collected by filtration to afford the desired compound as a white powder (2.57 g, 90%). **^{1}H NMR** (300 MHz, $CDCl_3$) δ 12.28 (s, 1H), 7.96 (d, *J* = 8.1 Hz, 1H), 7.87 (d, *J* = 7.9 Hz, 1H), 7.54 – 7.32 (m, 3H), 7.18 (d, *J* = 8.6 Hz, 1H), 7.01 (d, *J* = 8.5 Hz, 1H), 2.35 (s, 4H). **^{13}C NMR** (75 MHz, $CDCl_3$) δ 169.5, 155.9, 152.0, 133.8, 132.7, 128.7, 128.4, 126.7, 125.5, 122.2, 121.5, 117.7, 116.4, 20.5. **HLPC-MS (ESI):** calcd.: $C_{14}H_{11}NOS$ $[M]^+$: 241.1, found: 242.15.

3-(benzo[d]thiazol-2-*yl*)-2-hydroxy-5-methylbenzaldehyde. 2-(benzo[d]thiazol-2-*yl*)-4-methylphenol (2.57 g, 10.7 mmol, 1 equiv.) and hexamethylenetetramine (4.51 g, 32.0 mmol, 3.0 equiv.) were dissolved in TFA (18 mL) under inert atmosphere, the reaction was heated at reflux overnight. 4 M HCl (180 mL) was added to the reaction mixture and the resulting mixture was extracted with Et_2O (2 × 150 mL). The organic layer was dried wit $MgSO_4$, filtered, and the solvent evaporated. The crude was redissolved in cold Et_2O and to afford the desired compound as a white solid (1.28 g, 44%). **^{1}H NMR** (300 MHz, $CDCl_3$) δ 10.47 (s, 1H), 8.03 (d, *J* = 8.1 Hz, 1H), 7.93 (m, 2H), 7.70 (s, 1H), 7.54 (t, *J* = 7.2 Hz, 1H), 7.44 (t, *J* = 7.6 Hz, 1H), 2.40 (s, 3H).**^{13}C NMR** (75 MHz, $CDCl_3$) δ 191.2, 167.0, 158.7, 151.5, 135.4, 133.3, 132.9, 129.1, 127.0, 126.0, 123.8, 122.5, 121.7, 118.85, 20.4. **HLPC-MS (ESI):** calcd.: $C_{15}H_{11}NO_2S$ $[M]^+$: 269.1, found: 270.1.

***(E)*-4-(3-(benzo[d]thiazol-2-*yl*)-2-hydroxy-5-methylstyryl)-1-methylpyridin-1-ium (2).** 3-(benzo[d]thiazol-2-*yl*)-2-hydroxy-5-methylbenzaldehyde (1.00 g, 3.70 mmol, 1.0 equiv.), 1,4-dimethylpyridinium and piperidine (385.0 µL, 3.70 mmol, 1.0 equiv.) were dissolved in EtOH (75 mL) and refluxed overnight under N_2 atmosphere. The solvents were removed under vacuum and the resulting solid is redissolved in the minimal amount of MeOH and precipitated with cold Et_2O to afford the compound (**2**) after filtration as an orange solid (1.52 g, 85%). **^{1}H NMR** (300 MHz, DMSO) δ 13.05 (s, 1H), 8.84 (d, *J* = 6.9 Hz, 2H), 8.21 (d, *J* = 6.9 Hz, 3H), 8.16 – 8.04 (m, 2H), 7.76 (d, *J* = 9.6 Hz, 2H), 7.69 – 7.46 (m, 3H), 4.27 (s, 3H), 2.38 (s, 3H). **^{13}C NMR** (75 MHz, DMSO) δ 168.2, 154.0, 152.6, 150.7, 145.0, 134.7, 132.4, 130.6, 128.9, 127.1, 126.0, 124.3, 123.5, 123.4, 122.3, 121.9, 117.0, 46.9, 19.9. **HLPC-MS (ESI):** calcd.: $C_{22}H_{19}NOS^+$ $[M]^+$: 359.1, found: 359.2.

(*E*)-4-(3-(benzo[d]thiazol-2-yl)-5-methyl-2-(prop-2-yn-1-yloxy)styryl)-1-methylpyridin-1-ium (1). To a solution of compound **2** (972.0 mg, 2.0 mmol, 1.0 equiv.) in CH_3CN (7 mL) propargyl bromide (350. 0 µL, 4.0 mmol, 2.0 equiv.) and DIEA (700.0 µL, 4.0 mmol, 2.0 equiv.) were added, and the reaction mixture was refluxed overnight. Cold MeOH was added, and the precipitate was collected by filtration. Compound (**1**) was obtained as bright yellow solid after washing with MeOH (583.0 mg, 54%) [2]. **^{1}H NMR** (300 MHz, DMSO) δ 8.70 (d, *J* = 6.7 Hz, 2H), 8.06 (d, *J* = 7.1 Hz, 2H), 8.01 (s, 1H), 7.95 (d, *J* = 7.9 Hz, 1H), 7.91 – 7.80 (m, 2H), 7.68 (s, 1H), 7.46 – 7.31 (m, 2H), 7.26 (t, *J* = 7.7 Hz, 1H), 4.59 (s, 2H), 4.08 (s, 3H), 3.08 (s, 1H), 2.24 (s, 3H). **^{13}C NMR** (75 MHz, DMSO) δ 161.9, 152.2, 152.0, 151.7, 145.2, 135.5, 135.0, 133.9, 131.0, 130.6, 129.8, 127.4, 126.4, 125.5, 125.4, 123.9, 122.7, 122.0, 80.2, 78.1, 62.8, 47.0, 20.4. **HLPC-MS (ESI):** calcd.: $C_{25}H_{21}N_2OS^+$ $[M]^+$: 397.2 $[M+2H]^{2+}$: 199.2, found: 397.2, 199.2.

Spectroscopic characterization of 1/2

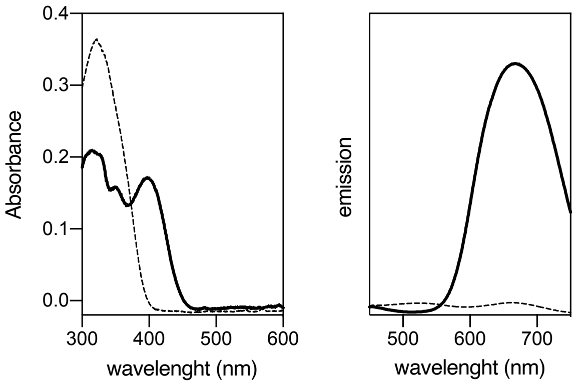

Figure S4. Left: Absorbance spectra of the caged probe **1** (dashed line) and the deprotected probe **2** (thick solid line). Right: fluorescence emission spectra **1** (dashed line) and **2** (thick solid line). Measurements taken at 20 μM, 7:3 v/v DMSO/water, λ_{exc} = 400 nm.

Circular Dichroism

Circular Dichroism experiments were made with a *Jasco-715* coupled with a *thermostat Nestlab RTE-111* with the following settings: Acquisition range: 300-195nm; band width: 2.0 nm; resolution: 0.2 nm; accumulation: 5 scans; sensitivity 10 mdeg; response time: 0.25 s, speed: 100 nm/min. The mixtures were incubated for 5 min before registering, and the spectra presented are the average of 5 scans.

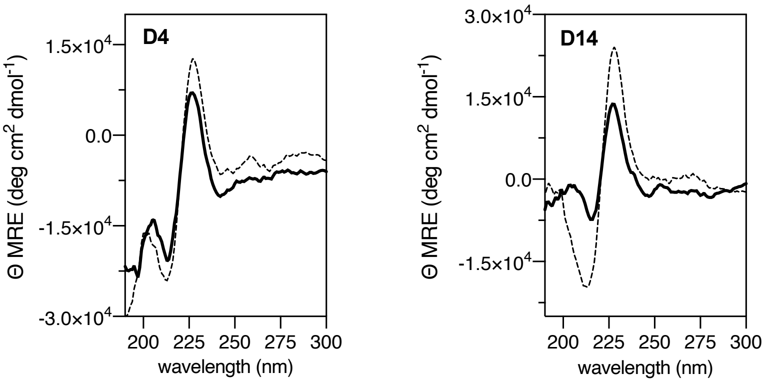

Figure S5. Left: CD spectra of 5 μM **D4** (dashed line) and 5 μM **D4** in the presence of and 1 equiv. of $PdCl_2$(COD) (thick solid line). Right: CD spectra of 5 μM **D14** (dashed line) and 5 μM **D14** in the presence of and 1 equiv. of $PdCl_2$(COD) (thick solid line). Measurements in 10 mM phosphate buffer pH 7.5 and 100 mM of NaCl using a 2 mm cell at 4 °C.

Residue correlation

Sequence logos are a standard method to display sequence patterns. The possible residues in each position are indicated, so that the height of each letter is proportional to its frequency in that position and the residues in each position are stacked so that the most frequent are placed at the top.

Logos were created using the WebLogo server (https://weblogo.berkeley.edu/).[4]

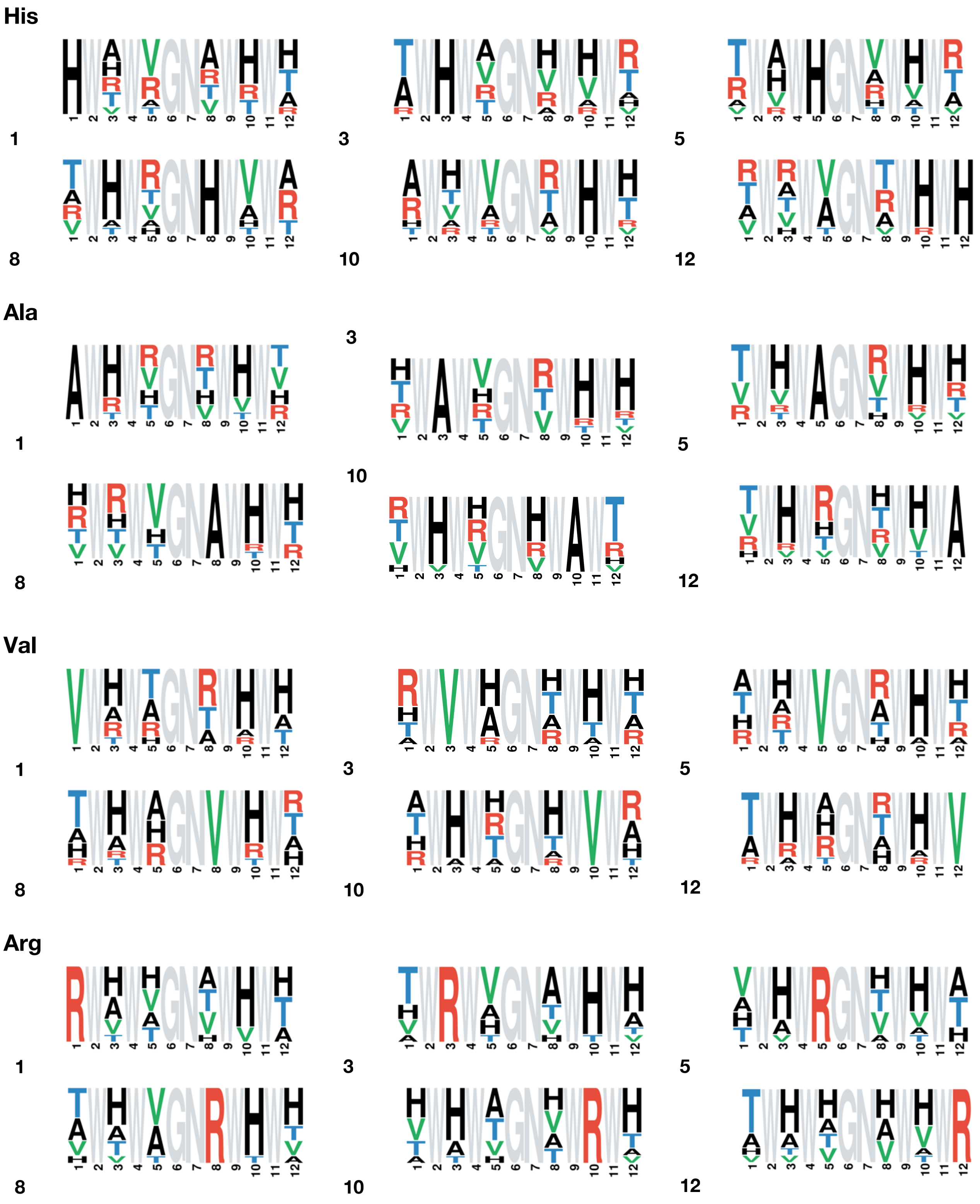

Figure S6. Each set of logos corresponds to the frequencies for each residue in all the screened positions (e.g., the first set corresponds to the frequencies when the His residue is at position 1, 3, 5, 8, 10, and 12. For clarity, the residues are color coded: Arg in red, Thr in blue, Val in green, His and Ala in black.

Thr

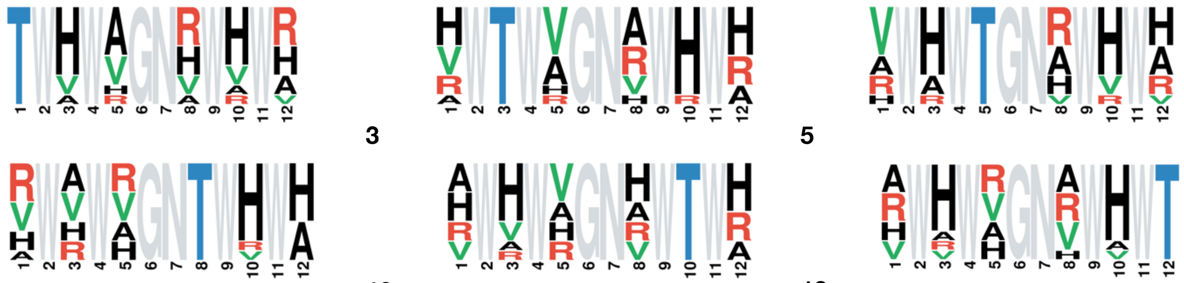

Figure S6. (cont.) Each set of logos corresponds to the frequencies for each residue in all the screened positions (e.g., the first set corresponds to the frequencies when the His residue is at position 1, 3, 5, 8, 10, and 12. For clarity, the residues are color coded: Arg in red, Thr in blue, Val in green, His and Ala in black.

Computational Studies

The optimization of the Pd-coordinated intermediates was carried out at the DFT level of theory using Gaussian16.[5] For the Pd, the B3LYP functional was used combined with the SDD pseudopotential and its associated double-ζ bases set as well as a set of *f* polarization functions;[6] for the rest of atoms, the same functional with the 6-31G(d) basis set was used, adding GD3 correction for dispersion.[7] Solvent was included with the solvent-polarizable dielectric continuum model (SMD) in water.[8] Harmonic frequency calculations were performed with tight convergence to ensure that the right vibrational modes were found.

Table S1. Relative energies of the complexes

Coord	ΔG(kcal)
cDD	0.00
cDE	0.14
cED	0.77
cEE	1.26
tDE	4.03
tDD	4.37
tED	4.71
tEE	5.13

The classical Molecular Dynamics were carried out with Amber20,[9] using the Amber14SB force field for the protein,[10] and the force constants and equilibrium parameters derived for Pd (from the above mentioned QM calculations) with the Seminario method. Point charges of Pd coordinated to both His were derived using RESP (Restrained Electrostatic Potential) model.[11] Finally, force field building was performed with MCPB.py.[12] The trajectories were set up with *xleap* (from AmberTools20), introducing each system in a cubic box of solvate TIP3P water molecules and Cl^- ions (*ions1lm_126_tip3p.lib*) to equilibrate charges. First, the (metallo)peptide is submitted to 3000 steps of minimization, then 100 ps of equilibration at constant volume followed by 500 ps of equilibration at constant pressure and finally 500 or 800 ns of production, all run with Amber20. Analysis of the trajectories was carried out with MDTraj library for Python.[13] The specific tools used include RMSD analysis along the trajectory, all-to-all RMSD analysis comparing each frame to the initial position, PCA and cluster counting to assess convergence of the simulation. Ramachandran plots were created using data from RamachanDraw for the density estimates;[14] ϕ and ψ angles from the whole trajectory were extracted with chimera. All plots were created with Matplotlib library for Python.[15]

In vitro studies

The catalytic deprotection of **1** to release the uncaged product **2** was performed in a 2.0 mL HPLC-vial with screw cap. For this purpose, fresh solution of **1** (10 μL, 20 mM in DMSO, 1.0 eq.) were to PBS (990 μL), and to the resulting mixture was added a solution of the palladium complex (1 μL, 20 mM in DMSO, 0.1 eq.). The reaction mixture was kept for 24 h at 37 °C under stirring at 1000 rpm. After that time, 50 μL of the reaction was taken and diluted to 100 μL with MeOH and analyzed by RP-HPLC-MS. The results were treated

according to the calibration curve, in which coumarin was used as internal standard. Every value is the average value of two independent measurements.

Biological experiments. General Information

All steps were performed on a sterile clean bench *Tesltar AV-100* at room temperature. Solutions stored in a fridge were warmed beforehand in a water bath (37 °C). Unless otherwise specified, all incubations were performed in FBS-DMEM (DMEM containing 5% of fetal bovine serum).

Cell Culture

All cell lines were cultured in DMEM (Dulbecco´s modified Eagle's medium), 5 mM glutamine, penicillin (100 units/mL) and streptomycin (100 units/mL) (all from Invitrogen). Proliferating cultures were maintained in a 5% CO_2 humidified incubator at 37 °C. For all the experiments, cells were seeded in the corresponding well at the indicated concentration two days before treatment.

Fluorescence microscopy

All images were obtained with an *Andor Zyla* mounted on a *Nikon TiE*. Confocal images were acquired in an *Andor Dragonfly High Speed Confocal Platform*. Images were further processed with *Image J* or *NIS software* (*Nikon*). Microscopy settings: The filter sets for the observation of the fluorescence of the products were as follows: **2**: Widefield: LED λ excitation: 385 nm. Filter cube: BP 375/28x nm, LP 515lp nm and DM 415 nm. **TMR**: Widefield: LED λ excitation: 550 nm. Filter cube TRITC-B-000 (Semrock): BP 543/22 nm, LP 593/40 nm and DM 562 nm. Confocal: Laser excitation: 561 nm. LP 620/60 and DM 567 nm.

Cell internalization studies

Cells were seeded on glass-bottom plates 48 h before treatment. Culture medium was removed and DMEM containing 5% fetal bovine serum (FBS-DMEM) and peptides (5 µM) or metallopeptides (5 µM) were added. Before the addition to cells, peptides were pre-incubated with metal complexes (1:1) in water for 10 min. After 30 min, cells were washed twice with PBS and replace with fresh FBS-DMEM to observe under the microscope with adequate filters. Digital pictures of the different samples were taken under identical conditions of gain and exposure.

Intracellular reactions

HeLa cells were seeded on glass-bottom plates 48 h before treatment. Culture medium was removed and FBS-DMEM containing probe **1** (50 µM) was added. After 1 h incubation, cells were washed twice with FBS-DMEM and a solution of metal complexes or metallopeptides (peptides were preincubated with metal complexes (1:1) in water for 10 min before the addition to cells) in FBS-DMEM were added. After a 1 h incubation, cells were washed twice in PBS and replace with fresh FBS-DMEM to observe under the microscope with adequate filters. Digital pictures of the different samples were taken under identical conditions of gain and exposure.

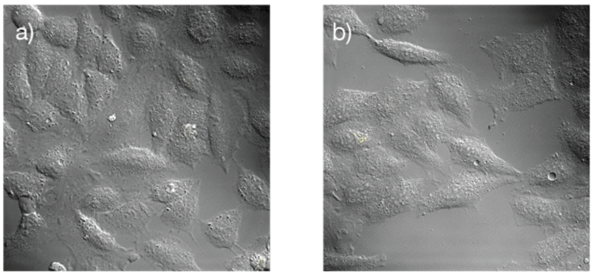

Figure S7. Control experiments of the intracellular depropargylation of **1** monitored by fluorescence microscopy in HeLa cells. a) Incubation for 1 h with the propargylated probe **1** at 50 µM concentration; b) Incubation with the palladium precursor $PdCl_2$(COD). Incubations were made in FBS-DMEM. Bright fields images are superimposed to orange emission channel recorded at 515–700 nm upon excitation at 385 nm.

ICP-MS

The metal content of the samples was determined by ICP-MS (*Agilent 7700x*) with a sample introduction system consisting of a *Micromist* glass low-flow nebulizer, a double-pass glass spray chamber with a Peltier system (3 ºC) and a quartz torch. For the ICP measurements, 100 000 cells/mL were seeded in 6-well plated two days before treatment with metallopeptide resulting from equimolar mixtures of **D4** or **D14** with $PdCl_2$(COD) or $PdCl_2$(COD), in FBS-DMEM for 1 h. Cells were then washed twice with PBS and lysed in 70% HNO_3. The lysates were digested in duplicate with HNO_3/H_2O_2 by MW heating before being analyzed.

HPLC MS product detection

HeLa cells were seeded in 6-well plate 48 h before the experiment. After this, first, the cells are incubated for 1 h with a 50 µM solution of the probe **1**. After washing twice with PBS, the cells are then incubated for 2 h with a 1:1 (50 µM) mixture of the peptide **D14** and $PdCl_2$(COD). After washing twice with PBS, 1 mL of MeOH is added to each well and the extracted contents of the cells are collected after 5 min. These solutions are concentrated at a rotary evaporator and re-dissolved in MeCN for HPLC-MS analysis in reverse phase.

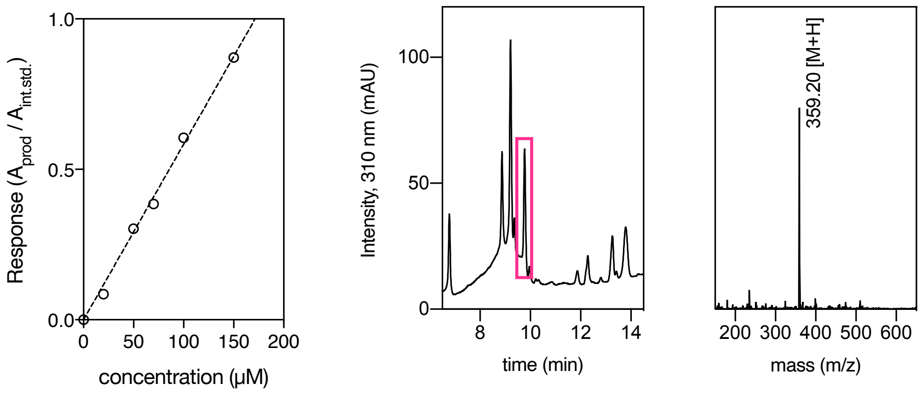

Figure S8. a) Calibration curve for compound **2**; b) reverse-phase HPLC-MS trace of the methanolic cell extracts of the intracellular reaction catalyzed with **D14**[Pd(II)], indicating the peak corresponding to the deprotected **2**; c) MS of the peak highlighted in b) with the mass of the depropargylated probe.

Viability test (MTT assay)

The toxicity of the peptide **D14**, the palladapeptide [Pd(II)]**D14**, and the $PdCl_2$(COD) salt were tested by MTT assay in HeLa cells.[15] 100 000 cells per well were seeded in 96 well plates two days before treatment with different concentrations of the compounds. After 24 h of incubation, HEPES containing 3-(4,5-dimethylthiazol-2-*yl*)-2,5-diphenyl tetrazolium bromide (MTT) was added to the cell culture medium to a final concentration of 0.5 mg/mL. Cells were then incubated for 4 h to allow the formation of formazan precipitates by metabolically active cells. A detergent solution of 10% sodium dodecyl sulphate (SDS) and 0.01 M HCl was then added, and the plate was incubated overnight at rt to allow the solubilization of the precipitates. The quantity of formazan in each well, which is directly proportional to the number of viable cells, was measured by recording changes in absorbance at 570 nm in a *Tecan Infinite F200 PRO* microtiter plate reading spectrophotometer.

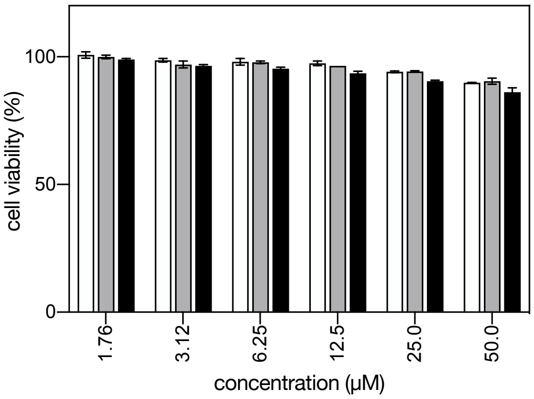

Figure S9. Toxicity assay. HeLa cells were incubated with **D14** (white bars), the palladapeptide [Pd(II)]**D14**, or the precursor salt $PdCl_2$(COD) at different concentrations, for 24 h at 37 °C. Finally, the cells were washed two times with PBS before carrying out the MTT assays. Data are represented as them mean +/- SEM for experimental repeated in three independent times. The palladapeptide [Pd(II)]**D14** was prepared by mixing the peptide **D14** with $PdCl_2$(COD) (1:1 ratio) in water for 10 min before the addition to cells.

Selection of the palladium precursor and preliminary catalysis asssays in PBS

Earlier studies conducted in our laboratory identified $PdCl_2$(COD) as the best palladium precursor in combination with bis-His α-helical peptides.[1] We confirmed that the β-hairpin TrpZip1 peptides identified in the SPOT library catalyzed the depropargylation more efficiently with $PdCl_2$(COD), followed by the bipyridine analog $PdCl_2$(bpy).

Preliminary catalytic assays in PBS with top-ranked peptides in the CelluSPOT assay (**D4**, **F14**, and **E9**) and their respective isomeric sequences that display low emission in the screening (**D4**, **F13**, **E9**). Note that $PdCl_2$(COD) palladium source is capable of effectively promoting the depropargylation reaction in PBS buffer in absence of peptide ligands.

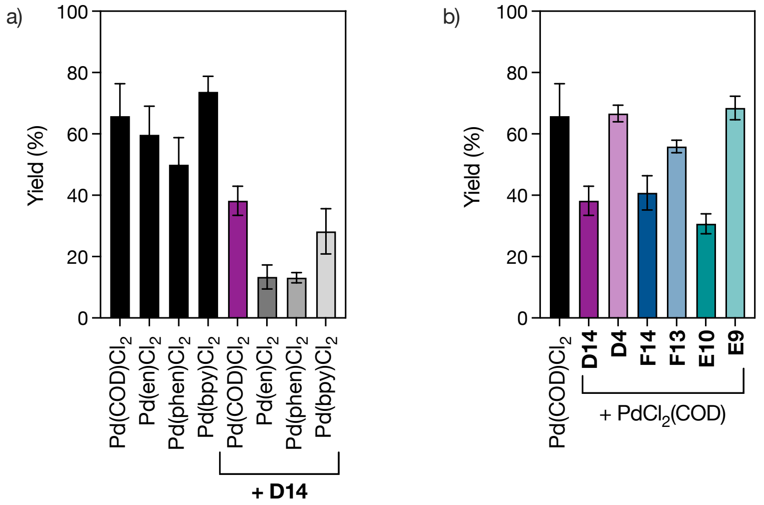

Figure S10. Left: *In vitro* catalysis of **D14** with different Palladium sources. Bar diagram representation of the yields obtained for each catalyst. Reaction conditions: 200 μM **1**, Pd source (20 μM, 10 mol%) in PBS at 37 °C, for 24 h. Yields were calculated by HPLC-MS(ESI) using internal standards. Right: *In vitro* catalysis of CelluSPOT peptides. Bar diagram representation of the yields obtained for each peptide in the presence of $PdCl_2$(COD). Reaction conditions: 200 μM **1**, 20 μM $PdCl_2$(COD), and 20 μM of each peptide in PBS at 37 °C, for 24 h.

Catalytic studies in DMAM, including additional hits from the SPOT library

Three of the top-ranked peptides, **F14**, **D14**, and **E10**, and their respective low-ranked isomeric sequences, **F13**, **D4**, and **E9**, were evaluated as $PdCl_2$(COD) ligands to promote the depropargylation of the fluorogenic probe **1**. The reactions were carried out in Dulbecco's Modified Eagle Medium (DMEM) instead of phosphate buffer, because DMEM significantly reduces the background catalysis of the palladium source observed in PBS (see Figure S10), providing a clearer assessment of the performance of each sequence.

a) Top hits CelluSPOT screen

rank	ID	1 3 5 8 10 12	% avg
1	F14	AWRWVGNTWHWH	99.1
2	D17	AWHWVGNRWHWT	93.0
3	D6	RWHWVGNAWHWT	92.0
4	H12	TWHWVGNHWAWR	91.3
5	D15	AWHWTGNRWHWV	89.4
6	F12	TWVWAGNRWHWH	86.0
7	B12	HWTWVGNAWHWR	85.0
8	D14	AWHWRGNVWHWT	84.6
9	H16	AWHWTGNHWVWR	84.5
10	F4	RWAWVGNTWHWH	84.4
11	H10	TWHWAGNHWVWR	83.4
12	E10	TWHWAGNVWRWH	82.8
13	F6	RWVWAGNTWHWH	81.7
14	F16	AWTWVGNRWHWH	77.3
15	J6	RWVWHGNAWHWT	75.2

b) control peptides

rank	ID	1 3 5 8 10 12	% avg
21	F13	AWRWTGNVWHWH	21.2
42	D4	RWHWAGNVWHWT	58.6
261	E9	TWHWAGNRWVWH	8.8

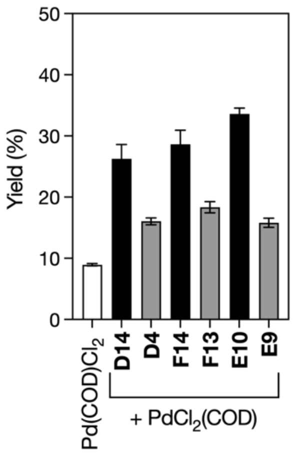

Figure S11. Left: a) best fifteen catalytic sequences ranked by their relative fluorescence emission intensity in the CelluSPOT screening after 24 h (green column); b) selected control sequences. Right: Bar diagram of the yields obtained for each catalyst in Dulbecco's Modified Eagle Medium (DMEM). Reaction conditions: 200 μM **1**, 100 μM $PdCl_2$(COD), and 100 μM of each peptide at 37 °C, for 24 h. Yields were calculated by HPLC-MS(ESI) using an internal standard.

2D NMR spectroscopy

To assess the presence of medium and long range interactions, we acquired 2D NOESY experiments of a 1 mM solution of **D14** (Figure S12, top), and in the presence of Pd(II) (Figure S12, top) shows good chemical shift dispersion and an increase in the number of NOEs, confirming the presence of side-chain interactions facilitated by the Pd coordination. For comparison, the spectra of free **D14** shows less dispersion and fewer NOEs. NMR spectra were acquired using a Bruker Avance III 600 spectrometer, at 298K, working at 600.23 MHz (^{1}H frequency), equipped with a z-pulse field gradient unit and a triple (^{1}H, ^{13}C, ^{15}N) resonance cryoprobe head. NOESY was acquired using a mixing time of 150 ms.[16,17] The data size in the time domain was 512 points in F1 (indirect dimension) and 2048 points in F2 (direct dimension). For each F1 value, 32 transients were accumulated in the NOESY. Data were processed with a combination of exponential and shifted sine-bell window functions for each dimension followed by automated baseline- and phase correction using *TopSpin* 3.5 (Bruker© 2020). The 512 × 2k data matrices were zero-filled to 2k × 2k (NOESY).

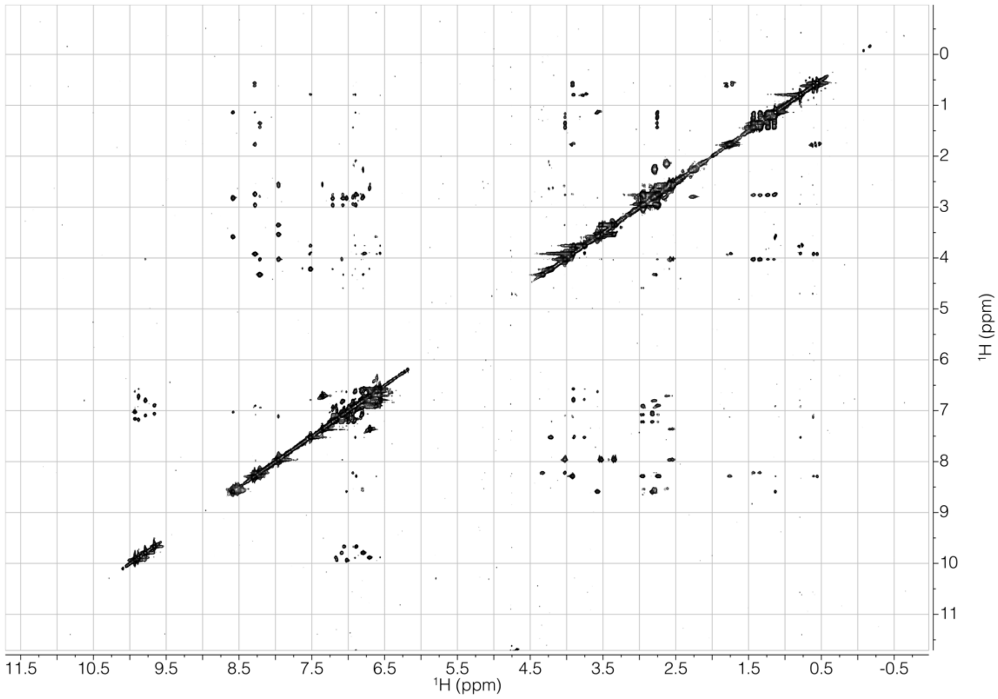

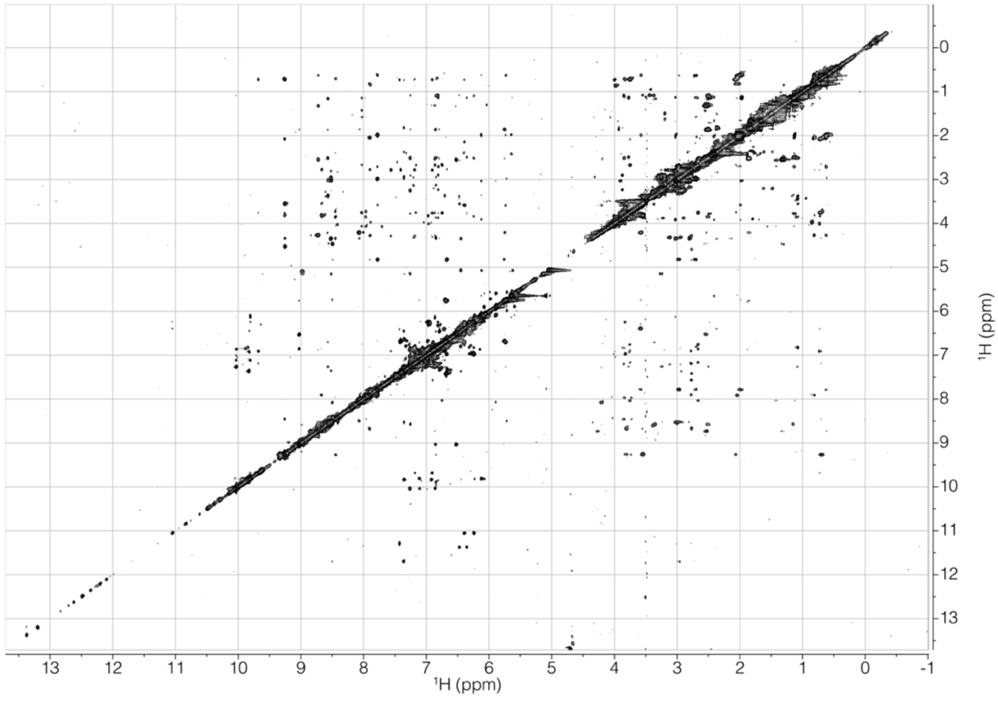

Figure S12. 2D NMR spectroscopy of a 1 mM solution of peptide **D14** (top) and the same solution of **D14** in the presence of 4.0 eq. $PdCl_2$(COD) (bottom).

Quantitative characterization of Pd(II) binding by D14 and D4

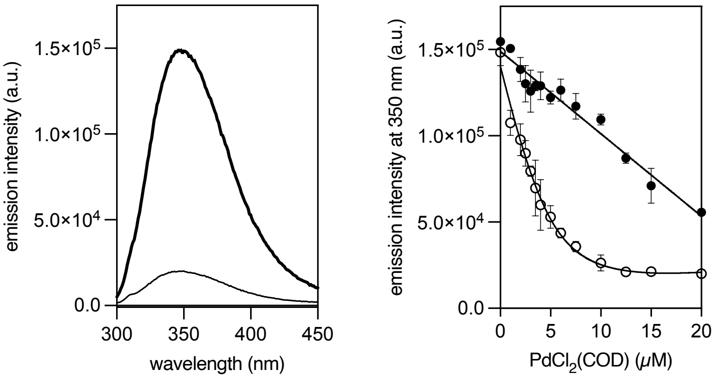

Figure S13. Pd(II) fluorescence titration of **D14** and **D4**. Left: fluorescence quenching of a 5 μM solution of **D14** in 25 mM HEPES buffer, NaCl 150 mM, pH 7.5 (thick line) and the same solution after incubation overnight with 4.0 eq. of Pd(II); Right: Tryptophan emission profile at 350 nm for 5 μM solutions of **D14** (white circles) and **D4** (black circles), incubated overnight with in increasing concentrations of $PdCl_2$(COD) and the best fit to 1:1 binding model. All the measurements were conducted in 25 mM HEPES buffer, 150 mM NaCl, pH = 7.5.

References

(1) Learte-Aymamí, S.; Vidal, C.; Gutiérrez-González, A.; Mascareñas, J. L. Intracellular Reactions Promoted by Bis(Histidine) Miniproteins Stapled Using Palladium(II) Complexes. *Angew. Chem. Int. Ed.* **2020**, *59* (23), 9149–9154.

(2) Gao, T.; Xu, P.; Liu, M.; Bi, A.; Hu, P.; Ye, B.; Wang, W.; Zeng, W. A Water-Soluble ESIPT Fluorescent Probe with High Quantum Yield and Red Emission for Ratiometric Detection of Inorganic and Organic Palladium. *Chem. Asian J.* **2015**, *10* (5), 1142–1145.

(3) Crooks, G. E.; Hon, G.; Chandonia, J.-M.; Brenner, S. E. WebLogo: A Sequence Logo Generator. *Genome Res.* **2004**, *14* (6), 1188–1190.

(4) Frisch, M. J.; Trucks, G. W.; Schlegel, H. B.; Scuseria, G. E.; Robb, M. A.; Cheeseman, J. R.; Scalmani, G.; Barone, V.; Petersson, G. A.; Nakatsuji, H.; Li, X.; Caricato, M.; Marenich, A. V.; Bloino, J.; Janesko, B. G.; Gomperts, R.; Mennucci, B.; Hratchian, H. P.; Ortiz, J. V.; Izmaylov, A. F.; Sonnenberg, J. L.; Williams; Ding, F.; Lipparini, F.; Egidi, F.; Goings, J.; Peng, B.; Petrone, A.; Henderson, T.; Ranasinghe, D.; Zakrzewski, V. G.; Gao, J.; Rega, N.; Zheng, G.; Liang, W.; Hada, M.; Ehara, M.; Toyota, K.; Fukuda, R.; Hasegawa, J.; Ishida, M.; Nakajima, T.; Honda, Y.; Kitao, O.; Nakai, H.; Vreven, T.; Throssell, K.; Montgomery, J. A., Jr; Peralta, J. E.; Ogliaro, F.; Bearpark, M. J.; Heyd, J. J.; Brothers, E. N.; Kudin, K. N.; Staroverov, V. N.; Keith, T. A.; Kobayashi, R.; Normand, J.; Raghavachari, K.; Rendell, A. P.; Burant, J. C.; Iyengar, S. S.; Tomasi, J.; Cossi, M.; Millam, J. M.; Klene, M.; Adamo, C.; Cammi, R.; Ochterski, J. W.; Martin, R. L.; Morokuma, K.; Farkas, O.; Foresman, J. B.; Fox, D. J. *Gaussian 16 Rev. B.01*; Wallingford, CT, 2016.

(5) Ehlers, A. W.; Böhme, M.; Dapprich, S.; Gobbi, A.; Höllwarth, A.; Jonas, V.; Köhler, K. F.; Stegmann, R.; Veldkamp, A.; Frenking, G. A Set of F-Polarization Functions for Pseudo-Potential Basis Sets of the Transition Metals Sc-Cu, Y-Ag and La-Au. *Chem. Phys. Lett.* **1993**, *208* (1), 111-114.

(6) Grimme, S.; Antony, J.; Ehrlich, S.; Krieg, H. A Consistent and Accurate Ab Initio Parametrization of Density Functional Dispersion Correction (DFT-D) for the 94 Elements H-Pu. *J. Chem. Phys.* **2010**, *132* (15), 154104.

(7) Marenich, A. V.; Cramer, C. J.; Truhlar, D. G. Universal Solvation Model Based on Solute Electron Density and on a Continuum Model of the Solvent Defined by the Bulk Dielectric Constant and Atomic Surface Tensions. *J. Phys. Chem. B* **2009**, *113* (18), 6378–6396.

(8) Case, {D A.; Belfon, K.; Ben-Shalom, I. Y.; Brozell, S. R.; Cerutti, D. S.; Cheatham, T. E.; Cruzeiro, V. W. D.; Darden, T. A.; Duke, R. E.; Giambasu, G.; Gilson, M. K.; Gohlke, H.; Goetz, A. W.; Harris, R.; Izadi, S.; Izmailov, S. A.; Kasavajhala, K.; Kovalenko, A.; Krasny, R.; Kurtzman, T.; Lee, T. S.; LeGrand, S.; Li, P.; Lin, C.; Liu, J.; Luchko, T.; Luo, R.; Man, V.; Merz, K. M.; Miao, Y.; Mikhailovskii, O.; Monard, G.; Nguyen, H.; Onufriev, A.; Pan, F.; Pantano, S.; Qi, R.; Roe, D. R.; Roitberg, A.; Sagui, C.; Schott-Verdugo, S.; Shen, J.; Simmerling, C. L.; Skrynnikov, N. R.; Smith, J.; J. Swails; Walker, R. C.; Wang, J.; Wilson, L.; Wolf, R. M.; Wu, X.; Xiong, Y.; Xue, Y.; York, D. M.; Kollman}, P. A. *AMBER 2020*; Univeristy of alifornia, San Francisco, 2020.

(9) Hornak, V.; Abel, R.; Okur, A.; Strockbine, B.; Roitberg, A.; Simmerling, C. Comparison of Multiple Amber Force Fields and Development of Improved Protein Backbone Parameters. *Proteins: Struct. Funct. Bioinf.* **2006**, *65* (3), 712–725.

(10) Bayly, C. I.; Cieplak, P.; Cornell, W.; Kollman, P. A. A Well-Behaved Electrostatic Potential Based Method Using Charge Restraints for Deriving Atomic Charges: The RESP Model. *J. Phys. Chem.* **1993**, *97* (40), 10269–10280.

(11) Li, P.; Merz, K. M. MCPB.Py: A Python Based Metal Center Parameter Builder. *J. Chem. Inf. Model.* **2016**, *56* (4), 599–604.

(12) McGibbon, R. T.; Beauchamp, K. A.; Harrigan, M. P.; Klein, C.; Swails, J. M.; Hernández, C. X.; Schwantes, C. R.; Wang, L.-P.; Lane, T. J.; Pande, V. S. MDTraj: A Modern Open Library for the Analysis of Molecular Dynamics Trajectories. *Biophys. J.* **2015**, *109* (8), 1528–1532.

(13) Cirilio, A. D. *RamachanDraw*. PyPI. https://pypi.org/project/RamachanDraw/ (accessed 2023-01-27).

(14) Hunter, J. D. Matplotlib: A 2D Graphics Environment. *Comput. Sci. Eng.* **2007**, *9* (3), 90–95.

(15) van Meerloo, J.; Kaspers, G. J. L.; Cloos, J. Cell Sensitivity Assays: The MTT Assay. *Methods Mol. Biol.* **2011**, *731*, 237–245.

(16) Macura, S.; Ernst, R. R. Elucidation of Cross Relaxation in Liquids by Two-Dimensional N.M.R. Spectroscopy. *Mol. Phys.* **1980**, *41* (1), 95–117.

(17) Wuthrich, K. *NMR of Proteins and Nucleic Acids*, 2nd ed.; Baker Lecture Series; Wiley-Blackwell: Chichester, England, 1996.
